# Khellactone Derivatives and Other Phenolics of *Phlojodicarpus sibiricus* (Apiaceae): HPLC-DAD-ESI-QQQ-MS/MS and HPLC-UV Profile, and Antiobesity Potential of Dihydrosamidin

**DOI:** 10.3390/molecules24122286

**Published:** 2019-06-19

**Authors:** Daniil N. Olennikov, Innokentii A. Fedorov, Nina I. Kashchenko, Nadezhda K. Chirikova, Cecile Vennos

**Affiliations:** 1 Institute of General and Experimental Biology, Siberian Division, Russian Academy of Science, 6 Sakh’yanovoy Street, Ulan-Ude 670047, Russia; ninkk@mail.ru; 2 Institute for Biological Problems of Cryolithozone, Siberian Division, Russian Academy of Science, 41 Lenina Street, Yakutsk 677000, Russia; fedorovia1958@mail.ru; 3 Department of Biochemistry and Biotechnology, North-Eastern Federal University, 58 Belinsky Street, Yakutsk 677027, Russia; hofnung@mail.ru; 4 Regulatory and Medical Scientific Affairs, Padma AG, 1 Underfeldstrasse, CH-8340 Hinwil, Switzerland; vennos_c@mail.ru

**Keywords:** *Phlojodicarpus sibiricus*, khellactone esters, dihydrosamidin, HPLC-MS, antiobesity activity, 3T3-L1 adipocytes

## Abstract

With obesity, the consumption of phenolic-enriched food additives as a part of traditional nutrition avoids the negative implications of eating high-calorie products. This study investigated the new herbal food additive, *Phlojodicarpus sibiricus* roots and herb, ubiquitously used in Siberia as a spice. Chromatographic techniques such as HPLC-DAD-ESI-QQQ-MS/MS and microcolumn HPLC-UV were the basic instruments for component profiling and quantification, and antiobesity potential was investigated using a differentiated 3T3-L1 adipocytes assay. We found that the roots and herb of *P. sibiricus* were high-coumarin-containing additives inhibiting triacylglycerol accumulation in 3T3-L1 preadipocytes. Forty-one phenolics were detected in *P. sibiricus* extracts, and 35 were coumarins, including 27 khellactone derivatives present as esters and glucosides. Total coumarin content varied from 36.16 mg/g of herb to 98.24 mg/g of roots, and from 0.32 mg/mL to 52.91 mg/mL in *P. sibiricus* preparations. Moreover, Siberian populations of *P. sibiricus* were characterised by a different HPLC-based coumarin profile. The most pronounced inhibiting effect on triacylglycerol accumulation in 3T3-L1 preadipocytes was shown for dihydrosamidin (khellactone 3′-*O*-isovaleroyl-4′-*O*-acetyl ester), which was more active than other khellactone esters and glucosides. The results demonstrated that if used as a food additive *Phlojodicarpus sibiricus* could be a source of bioactive coumarins of the khellactone group with high antiobesity potential.

## 1. Introduction

The pathological condition of obesity is generally characterised as an energy imbalance due to increased consumption of high-calorie foods, especially high-lipid-containing products. Additional factors, but not less important, are economic and social issues as well as hypodynamia and a high level of overall food consumption [1]. To reduce the risk factors associated with obesity, various methods of prevention and treatment are used, including the frequent use of pharmacotherapy that improves the patient’s condition. The disadvantages of traditional synthetic drugs used to treat obesity are low effectiveness, a narrow therapeutic range, toxicity and significant side effects. Because of this, increased attention has been paid to plant-based medicines, which are devoid of most of these disadvantages of synthetic drugs [2].

During the course of long-term investigations, the effectiveness of plant medicines containing and concentrating various groups of phenolic compounds has been proven. Species of the Apiaceae family are a promising plant source of phenolics possessing antiobesity activity [3]. Among the many representatives of this family, the genus *Phlojodicarpus*, a small genus of the Apiaceae family with four species—*P. sibiricus* (Fisch.) Koso-Pol., *P. turczaninovii* Sipliv., *P. villosus* (Turcz. ex Fisch. & C.A. Mey.) Turcz. ex Ledeb., and *P. popovii* Sipliv.—should be particularly noted [4]. The geographic distribution of *Phlojodicarpus* species includes the territories of Siberia, the Far East and less frequently Mongolia, where this genus is common in steppe and near-forest communities [4]. The scientific interest in species of *Phlojodicarpus* is due to the presence of coumarins, marker components of the Apiaceae family [5]. Three *Phlojodicarpus* species (excluding *P. popovii*) were chemically investigated as sources of coumarins; among them, 31 compounds of the simple group, furocoumarins and pyranocoumarins, were isolated [6,7,8,9,10,11,12,13,14,15] (Table S1). Insufficient attention has been given however to the other groups of phytocomponents.

**Figure 1:**
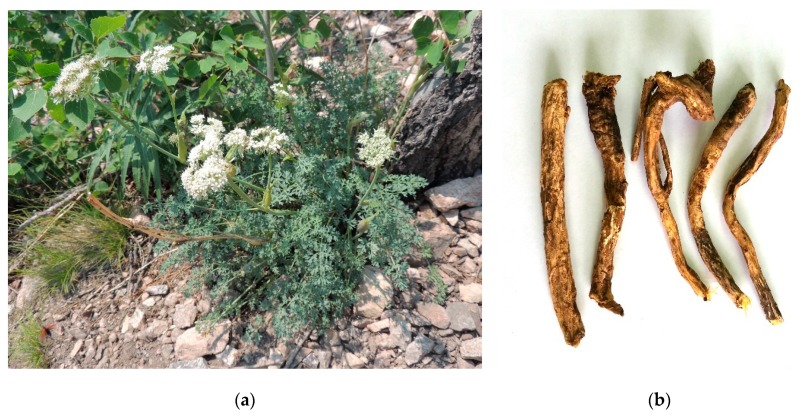
*Phlojodicarpus sibiricus* (Fisch.) Koso-Pol. plant in nature habitat (**a**); Siberia, Buryatia Republic and its roots (**b**).

Among these species, *P. sibiricus* is most prevalent in Siberia and Yakutia (Figure 1). This species was widely used by most nomadic and sedentary peoples of Siberia as a food and medicinal plant. Fragrant powder from the roots and herb of *P. sibiricus*, alone or mixed with salt, was used as a spice for mutton and horsemeat [16]. The mixture of ground roots and seeds is still used as a flavouring agent to improve the taste of mutton broth in Siberia [17]. Yakut healers (*otosut*) and shamans used mainly the roots of *P. sibiricus* (*бѳрѳ сиир oтo*, ‘boro siir oto’) as a decoction for obesity, pulmonary tuberculosis, diseases of the thyroid gland, stomach and heart, and rheumatism, as well as for wounds and tumours of the stomach and oesophagus [18]. Buryat lamas, also called *ru-rta* (the roots of *P. sibiricus*, less commonly the herb) was used to treat diseases of the blood, lungs, stomach, and throat [19]. A similar application for *P. sibiricus* has been identified in Mongolian medicine [20]. The tincture of the whole plant in vodka (40% ethanol) was recommended by healers in the Zabaykalsky Krai of Siberia for pathologies such as obesity and atherosclerosis [21]. According to preliminary pharmacological investigations, the extract of the roots of *P. sibiricus* showed spasmolytic, coronary, and pronounced anti-atherosclerotic benefits [22].

The known literature data on the chemical composition of the roots of *P. sibiricus* refers only to plants growing in the Far East, and mentions visnadine [6], dihydrosamidine [7], umbelliferone and scopoletin [8]. Suksdorfin, khellactone 4′-*O*-methyl ester and khellactone 3′-*O*-acetyl-4′-*O*-(2-methylbutyroyl) ester [9] were discovered among the components of the *P. sibiricus* herb from the Zabaykal’sky Krai; plants from Mongolia were found to accumulate phlojodicarpin, isophlojodicarpin [10], umbelliferone 7-*O*-(6′-apiofuranosyl)-glucopyranoside (6′-apiosylskimmin), 8-(2′,3′-dihydroxy-3′-methylbutyl)oxycoumarin 7-*O*-glucopyranoside and diosmetin 7-*O*-glucopyranoside [11]. Suksdorfin, khellactone 3′-*O*-acetyl-4′-*O*-(2-methylbutyroyl) ester, 6′-apiosyl-skimmin and isoimperatorin [12] were identified in the herb of *P. sibiricus* from the northern populations of Russia. We also know about the fatty acid composition of *P. sibiricus* roots from some Siberian populations [23].

Recently, considerable attention has been paid to khellactone esters and their plant sources due to their potential for high effectiveness as antiadipogenic agents [24]. Plant extracts from Apiaceae family members such as *Peucedanum praeruptorum*, *P. decursivum* and *P. japonicum* previously demonstrated the ability to reduce obesity and diabetes symptoms in animals fed high-fat diets [25]. Regarding the positive influence of these plants on diseased animals, it was found that such lipophilic components as the dihydropyranocoumarins of the khellactone group can exert an inhibitory effect on adipogenesis both in vitro and in vivo [3]. It was also shown that within the botanical genus, plants with and without antiadipogenic effectiveness may exist [26]. Considering the ethnopharmacological data on the use of *P. sibiricus* as an anti-obesity remedy, as well as data on the presence of dihydropyranocoumarins in this plant, it was of interest to study the connection of these facts. This would enable us to identify the chemical basis for the biological efficacy of *P. sibiricus* preparations and to determine the active component with the highest efficacy.

It should also be noted that this plant species has not previously been submitted to chromatographic profiling. Therefore, one cannot speculate about the chemical diversity of the components of the metabolome and determine the prospects for its further research. The lack of quantitative data on the content of coumarins in different parts of *P. sibiricus* makes it impossible to create dosage forms that are effective and enriched with active substances. Thus, to investigate the ethnopharmacological validity of arguments in favour of *P. sibiricus* use as an anti-obesity remedy, we first performed chromatographic profiling of *P. sibiricus* roots and herb using the HPLC-DAD-ESI-QQQ-MS/MS technique, followed by quantification of basic components with the HPLC-UV methodology. Then we examined the inhibitory effects of selected compounds on triacylglycerol accumulation in differentiated 3T3-L1 adipocytes.

## 2. Results

### 2.1. Chemical Composition of Phlojodicarpus sibiricus Organs and Their Influence on Triacylglycerol Content in 3T3-L1 Preadipocytes

Apiaceous plants are known for their ability to accumulate various groups of natural compounds such as essential oil (as in *Ferula* [27]), coumarins (as in *Peucedanum* [28] or *Angelica* [29]), flavonoids and phenylpropanoids (as in *Cumin* [30] or *Foeniculum* [31]) and polysaccharides (as in *Daucus* [32]). Chemical components of *Phlojodicarpus sibiricus* are a mixture of lipophilic and hydrophilic compounds. Roots of *P. sibiricus* are the concentrators of essential oil (7.52 mg/g), coumarins (108.94 mg/g), caffeoylquinic acids (18.62 mg/g), water-soluble polysaccharides (3.74 mg/g) and pectic substances (2.61 mg/g) (Table 1). The components in seeds of *P. sibiricus* were in trace or low level. Flavonoid content in roots, herb and seed was low.

Using a 3T3-L1 preadipocytes differentiation assay, we identified inhibitory activity of the extracts from *P. sibiricus* roots and herb on the triacylglycerol accumulation (Table 1). Extract of *P. sibiricus* roots at doze 50 μg/mL decreased the level of triacylglycerol compared to 526.4 μg/mg, with the control group at 812.8 μg/mg. Herb extract was a less effective inhibitor, and activity of seed extract was insignificant. Various lipophilic fractions of the extract of *P. sibiricus* roots demonstrated a hexane fraction as the most active and a water fraction as intermediately active (Table S2). Essential oil of *P. sibiricus* roots, containing sabinene and limonene as the main components, had weak activity (Table S3). Water-soluble polysaccharides and pectic substances with a high content of glucose/galactose and uronic acids, respectively, were inactive fractions of *P. sibiricus* roots (Table S4).

In theory, coumarins as basic extracts of *P. sibiricus* roots should have the greatest bioactivity. Regression analysis demonstrated good linearity between the total coumarin content in roots of 12 *P. sibiricus* samples and their inhibitory activity on the triacylglycerol accumulation in 3T3-L1 preadipocytes (Figure S1). Given the known information that dihydropyranocoumarins possess antiobesity potential [3], coumarins of *P. sibiricus* might be extensively studied as the prospective natural biocomponents.

### 2.2. Khellactone Derivatives and Other Coumarins as the Main Phenolics of P. sibiricus: HPLC-DAD-ESI-QQQ-MS/MS Profile of Root and Herb Extracts

**Figure 2:**
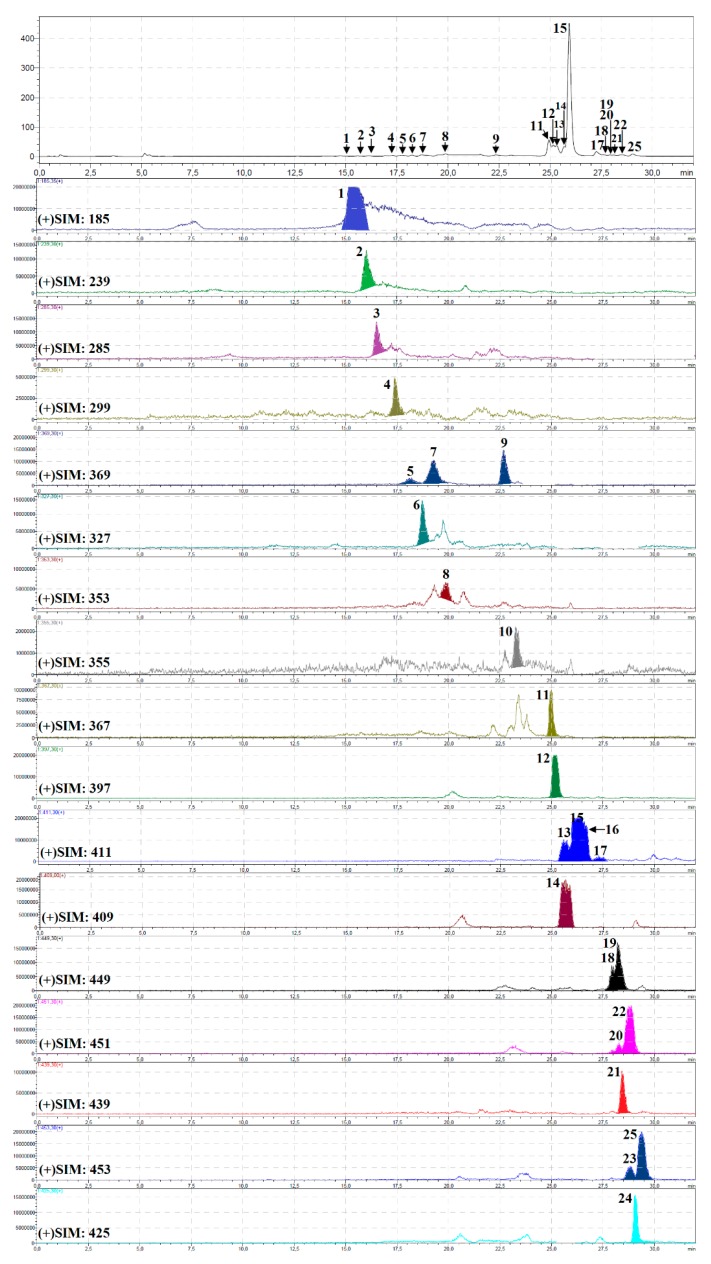
HPLC-DAD chromatogram (330 nm); (**a**) and HPLC-ESI-MS chromatograms selected ion monitoring mode (SIM), positive ionization; (**b**) of hexane fraction of *Phlojodicarpus sibiricus* roots. Compounds are numbered as listed in Table 2.

**Figure 3:**
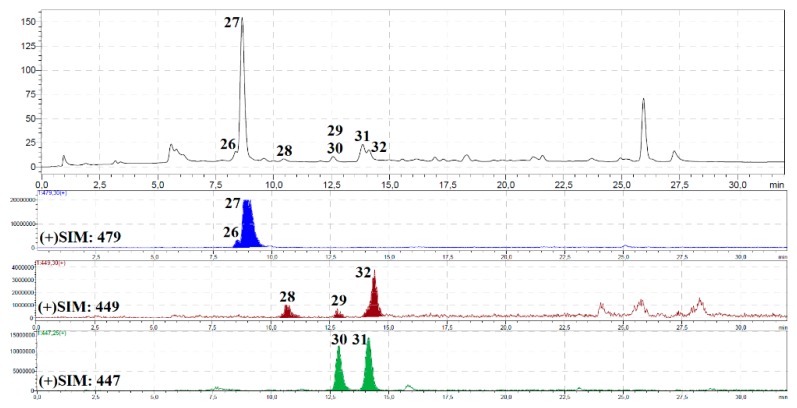
HPLC-DAD chromatogram (330 nm); (**a**) and some HPLC-ESI-MS chromatograms SIM mode, positive ionization; (**b**) of methanol fraction of *P. sibiricus* roots. Compounds are numbered as listed in Table 2.

**Figure 4:**
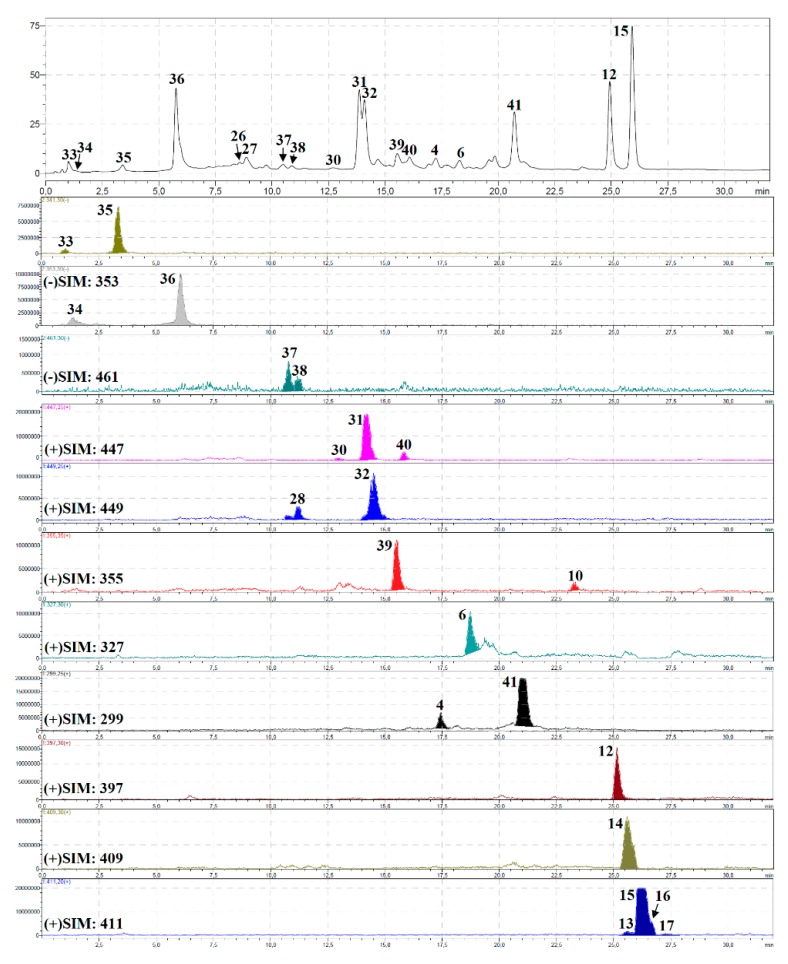
HPLC-DAD chromatogram (330 nm; (**a**) and some HPLC-ESI-MS chromatograms (SIM mode, positive/negative ionization; (**b**) of methanol extract of *P. sibiricus* herb. Compounds are numbered as listed in Table 2.

The component profile of *P. sibiricus* roots and herb extracts was recorded by high performance liquid chromatography with diode array detection and a triple quadrupole electrospray ionisation detector (HPLC-DAD-ESI-QQQ-MS/MS) in both positive and negative modes. The identification results are summarised in Table 2; chromatograms of the hexane and methanol fractions of *P. sibiricus* roots and methanol extract of *P. sibiricus* herb are presented as Figure 2, Figure 3 and Figure 4. Twenty-five pure compounds were used as references for identification, and their structures are shown in Figure S3.

#### 2.2.1. Khellactone and It Esters

The coumarin khellactone {9,10-dihydro-9,10-dihydroxy-8,8-dimethyl-2*H*,8*H*-benzo(1,2-b:3,4-b′) dipyran-2-one}, related to the pyranocoumarins group, is widely distributed both in free state and in the form of ethers in members of the Apiaceae family including *Angelica* [29], *Peucedanum* [28], *Phlojodicarpus* [9], *Seseli* [33] species and others. In the hexane fraction of the roots of *P. sibiricus* growing in Yakutia, khellactone (**3**) and 21 derivatives of **3** (compounds **4**–**7**, **9**–**25**) were detected and identified as *O*-ethers of khellactone in positions 3′ and 4′, where khellactone contains hydroxyl groups. Khellactone and its *O*-ethers were characterised by a close UV pattern with a maximum in the area of 322 ± 2 nm (Figure S2) and specific mass-spectral behavior.

**Figure 5:**
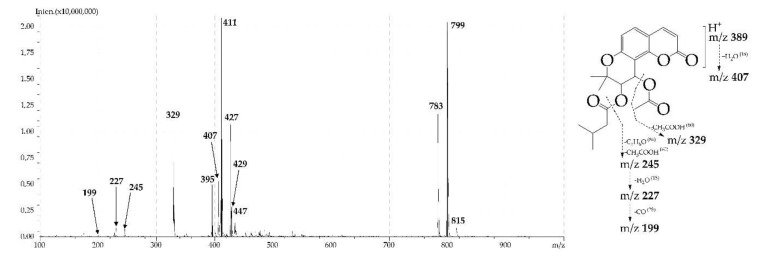
ESI-MS spectrum (positive ionization) and fragmentation path of khellactone 3′-*O*-isovaleroyl-4′-*O*-acetyl ester (dihydrosamidin, **15**).

Using an example of a 3′,4′-disubstituted derivative of khellactone **15** (khellactone 3′-*O*-isovaleroyl-4′-*O*-acetyl ester or dihydrosamidin), we consider the features of fragmentation of khellactone ethers under ESI-MS in positive ionisation mode (Figure 5). In the mass spectrum of **15**, the intensity of the ion of the protonated molecule [M + H]^+^ with *m*/*z* 389 was very low (no more than 1%). The most intense signals were caused by adduct ions with Na^+^ (*m*/*z* 411) and K^+^ (*m*/*z* 427). It is interesting to note the presence of a signal of adduct with Li^+^ ions (*m*/*z* 395), rarely described for natural coumarins.

The protonated fragment [M + H]^+^ gave a more intense signal of the hydrated ion [(M + H) + H_2_O]^+^ with *m*/*z* 407, as well as the fragment of the adduct [M + Na]^+^ forming the ion [(M + Na) + H_2_O]^+^ with *m*/*z* 429. Fragmentation of the main skeleton of molecule **15** occurred as a result of the successive removal of substituents from positions 4′ and 3′ from the protonated fragment [M + H]^+^.

In the beginning, acetic acid was removed from the 4′-*O*-acetyl fragment, as indicated by the presence of a signal with *m*/*z* 329; then a 3′-*O*-isovaleroyl fragment of C_5_H_8_O was removed, forming a fragment with *m*/*z* 245 [(M + H) – C_2_H_4_O_2_ – C_5_H_8_O]^+^. It should be noted that the cleavage of the 4′-*O*-acyl substituent occurred with the cleavage of the C4′-*O* bond, while at the removal of the 3′-*O*-acyl fragment the C3′-*O* bond remained. The elimination of H_2_O and CO molecules from the deacylated molecule with the formation of fragments with *m*/*z* 227 and 199, respectively, was the final process. The features of the spectrum of **15** include the easy formation of dimeric fragments with Li^+^, Na^+^ and K^+^ ions, which form a specific triad of signals with *m*/*z* 783 [2M + Li]^+^, 799 [2M + Na]^+^ and 815 [2M + K]^+^. In general, the described mass spectral pattern was observed for all *O*-esters of khellactone. The differences related to the intensity of individual signals and the presence or absence of certain ions of the adducts, as well as signals from the protonated fragment.

To facilitate the identification of components, reference compounds were used to identify twelve substances, including khellactone 4′-*O*-methyl ester (**4**), khellactone 4′-*O*-acetyl ester (**6**), khellactone 3′,4′-di-*O*-acetyl ester (**9**), khellactone 4′-*O*-angeloyl ester or d-laserpitin (**11**), khellactone 3′-*O*-acetyl-4′-*O*-isobutyroyl ester or hyuganin D (**12**), khellactone 3′-*O*-(2-methylbutyroyl)-4′-*O*-acetyl ester or visnadin (**13**), khellactone 3′-*O*-acetyl-4′-*O*-angeloyl ester or pteryxin (**14**), khellactone 3′-*O*-isovaleroyl-4′-*O*-acetyl ester or dihydrosamidin (**15**), khellactone 3′-*O*-acetyl-4′-*O*-isovaleroyl ester or suksdorfin (**16**), khellactone 3′-*O*-acetyl-4′-*O*-(2-methylbutyroyl) ester or hyuganin C (**17**), khellactone 3′,4′-di-*O*-senecioyl ester (**18**) and khellactone 3′,4′-di-*O*-angeloyl ester or praeruptorin D (**19**). Of the compounds mentioned, khellactone 4′-*O*-methyl ester (**4**) [9], hyuganin D (**12**) [9], visnadin (**13**) [6], dihydrosamidin (**15**) [7] and suksdorfin (**16**) [9] were previously found in *P. sibiricus*, while compounds **6**, **9**, **11**, **14** and **17**–**19** were identified for the first time in this species.

Compounds **5** and **7** gave adduct ions with *m*/*z* 347 [M + H]^+^, suggesting they were isomers and represented the 4′-*O*-monosubstituted ester of khellactone with an isovaleroyl or isomeric 2-methylbutyroyl substituent. The location of the substituent at C-4′ was indicated by loss of a fragment with *m*/*z* 102 (C_5_H_10_O_2_), which is formed when the C4′-*O* bond is broken. The presence of a substituent at C-3′ would lead to the removal of a fragment with *m*/*z* 84 (C_5_H_8_O), keeping the C3′-*O* bond; this species was not detected. Thus, the structure of compounds **5** and **7** can be described as khellactone 4′-*O*-isovaleroyl ester or khellactone 4′-*O*-2-methylbutyroyl ester. Khellactone 4′-*O*-isovaleroyl ester was previously isolated from *Lomatopodium staurophyllum* (Rech.f.) Rech.f. (Apiaceae) [34], and khellactone 4′-*O*-2-methylbutyroyl ester was detected in *Peucedanum japonicum* Thunb. (Apiaceae) [35]. None of the mentioned compounds was previously identified in *P. sibiricus*.

The mass spectrum of compound **10** gave a signal of the adduct ion with *m*/*z* 355 [M + Na]^+^, which is 14 a.m.u. (CH_2_) smaller than in compounds **5** and **7**, indicating that it is an *O*-isobutyroyl ester of khellactone. The mass of the leaving fragment C_4_H_8_O_2_ (*m*/*z* 88) confirmed its attachment at the position C4′ [36]. Thus, compound **10** was khellactone 4′-*O*-isobutyroyl ester. Although disubstituted derivatives of khellactone with the isobutyroyl group in the C4′ position are found in Apiaceae species, khellactone 4′-*O*-isobutyroyl ester itself has not been previously found in this family.

For compounds **20** and **22**, the presence of protonated ion [M + H]^+^ with *m*/*z* 429 and adduct ions with *m*/*z* 451 [M + Na]^+^ and 467 [M + K]^+^ was noted in the mass spectra. Despite a close set of signals in the spectra of **20** and **22**, one significant difference was observed. Compound **20** had a signal with *m*/*z* 329 (senecic acid or angelic acid), indicating that the C_5_H_8_O_2_ fragment was cleaved from C4′; the mass spectrum of compound **22** contained a signal with *m*/*z* 327, indicating the removal of a C_5_H_10_O_2_ fragment (isovaleric acid or 2-methylbutyric acid) from the same position. The presence of a common signal *m*/*z* 245 for derivatives of khellactone indicated that in compound **20** at position C3′ a fragment C_5_H_8_O (isovaleroyl or 2-methylbutyroyl) was removed, and in compound **22** a fragment C_5_H_6_O (senecioyl or angeloyl) was removed. The presented data make it possible to establish the structure of compound **20** as khellactone 3′-*O*-isovaleroyl/2-methylbutyroyl-4′-*O*-senecioyl/angeloyl ester, and compound **22** is khellactone 3′-*O*-senecioyl/angeloyl-4′-*O*-isovaleroyl/ 2-methylbutyroyl ester, which is isomeric to compound **20**. There are four possible structures for each of these two compounds. Khellactone 3′-*O*-isovaleroyl-4′-*O*-angeloyl ester, 3′-*O*-isovaleroyl-4′-*O*-senecioyl ester, khellactone 3′-*O*-2-methylbutyroyl-4′-*O*-angeloyl ester, khellactone 3′-*O*-2-methylbutyroyl-4′-*O*-senecioyl ester, khellactone 3′-*O*-senecioyl-4′-*O*-isovaleroyl ester and khellactone 3′-*O*-senecioyl-4′-*O*-2-methylbutyroyl ester were detected in *Peucedanum japonicum* [35,37], and khellactone 3′-*O*-angeloyl-4′-*O*-isovaleroyl ester was detected in *Seseli tortuosum* L. (Apiaceae) [33]. Compounds with similar structures in *P. sibiricus* were not identified previously.

Compound **21** produced in the mass spectrum a protonated ion with *m*/*z* 417 and fragmentation products with *m*/*z* 329 [(M + H) – C_4_H_8_O_2_]^+^ and *m/z* 245 [(M + H) – C_4_H_8_O_2_ – C_5_H_8_O]^+^, indicating sequential removal of isobutyric acid and an isovaleroyl (or 2-methylbutyroyl) fragment, respectively. A compound possessing a similar decomposition may have the structure of 3′-*O*-2-methylbutyroyl-4′-*O*-isobutyroyl ester (previously isolated from *Peucedanum japonicum* [35]) or khellactone 3′-*O*-isovaleroyl-4′-*O*-isobutyroyl ester (which is not yet found in nature).

Compounds **23** and **25** had the same set of bands in the mass spectrum, including a weak protonated ion [M + H]^+^ with *m*/*z* 431 and intense ion adducts with *m*/*z* 453 [M + Na]^+^ and 469 [M + K]^+^. Deacylation at position C4′ led to the removal of the C_5_H_10_O_2_ fragment (isovaleric acid or 2-methylbutyric acid) and the formation of a fragment with *m*/*z* 329. In the second stage, the C_5_H_8_O fragment (isovaleroyl or 2-methylbutyroyl) was separated from the molecule to form a fragment with *m*/*z* 245. Four compounds correspond to this type of decomposition: khellactone 3′,4′-di-*O*-isovaleroyl ester found in *Seseli tortuosum* [33], khellactone 3′,4′-di-*O*-2-methylbutyroyl ester, khellactone 3′-*O*-isovaleroyl-4′-*O*-2-methylbutyroyl ester (praeruptorin H) from *Peucedanum praeruptorum* Dunn (Apiaceae) [38] and the still unknown khellactone 3′-*O*-2-methylbutyroyl-4′-*O*-isovaleroyl ester.

Compound **24** gave the ion [M + H]^+^ with *m*/*z* 403 and two other ions, with *m*/*z* 315 [(M + H) – C_4_H_8_O_2_]^+^ and *m*/*z* 245 [(M + H) – C_4_H_8_O_2_ – C_4_H_6_O]^+^, indicating the removal of the same isobutyric fragments. Thus, compound **24** was khellactone 3′,4′-di-*O*-isobutyroyl ester, recently found in *Glehnia littoralis* F.Schmidt ex Miq. (Apiaceae) [39].

Compounds **4**, **6**, **12** and **15**, as well as the two components **39** and **41** (absent in the roots) were found in the methanol extract of the *P. sibiricus* herb. In the mass spectrum of trace compound **39**, only adduct ions with *m*/*z* 355 [M + Na]^+^ and 371 [M + K]^+^, characteristic of the khellactone *O*-isobutyroyl ester, were noted. Compound **41** contained in the mass spectrum bands of adduct ions with *m*/*z* 299 [M + Na]^+^ and 315 [M + K]^+^, as well as a weak ion of the demethylated fragment with *m*/*z* 263 [(M + H) – CH_2_]^+^. A similar picture of decomposition is characteristic for monomethyl derivatives of the khellactones. One in particular, khellactone 4′-*O*-methyl ester (**4**), has already been identified in the roots and herb of *P. sibiricus*. Therefore, the most likely structure corresponding to compound **41** may be khellactone 3′-*O*-methyl ester.

#### 2.2.2. Khellactone Glucosides

**Figure 6:**
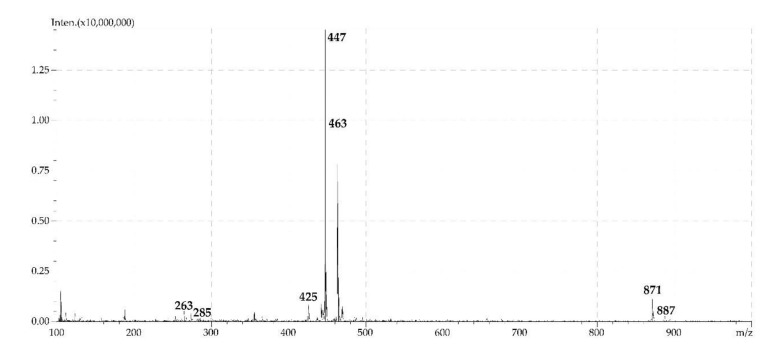
ESI-MS spectrum (positive ionization) of khellactone-3′-*O*-glucoside (praeroside II, **31**).

Khellactone glucosides were detected in the roots (compounds **30** and **31**) and the herb of *P. sibiricus* (compounds **30**, **31**, and **40**). Using a reference compound, the presence of khellactone 3′-*O*-glucoside or praeroside II (**31**) was established. In the mass spectrum of **31**, a weak band of protonated ion [M + H]^+^ with *m*/*z* 425 and intense bands of adduct ions with *m*/*z* 447 [M + Na]^+^ and 463 [M + K]^+^ were observed (Figure 6). As a result of deglucosylation, fragments with *m/z* 285 [(M + Na)–Glc]^+^ and *m*/*z* 263 [(M + H) – Glc]^+^ were formed. As for khellactone *O*-esters, easy formation of dimers was observed with *m*/*z* 871 [2M + Na]^+^ and 887 [2M + K]^+^. Compound **30,** with a lower chromatographic mobility, was characterised by a close mass spectrum and was identified as an isomeric khellactone 4′-*O*-glucoside. The same can be noted for trace component **40**, which is defined as khellactone *O*-hexoside due to lack of more information. The presence of khellactone glucosides has not been established previously for the genus *Phlojodicarpus*, however, their presence has been shown in the species *Angelica* [40] and *Peucedanum* [41].

#### 2.2.3. Coumarins with Various Structures

Simple coumarins **1** and **2** were easily identified as the known plant components umbelliferone and bergapten, respectively, using reference compounds.

In the roots of *P. sibiricus*, the mass spectrum of compound **8** gave a protonated ion [M + H]^+^ with *m*/*z* 331 and mass spectral pattern close to that of compound **5**, but with 16 a.m.u. less. Such behaviour is suggestive of a deoxy analogue of the khellactone lomatin, thus indicating structure **8** is lomatin *O*-isovaleroyl ester as detected in *Haplophyllum kowalenskyi* Stschegl. (Rutaceae) [42], or an unknown lomatin *O*-2-methylbutyroyl ester.

**Figure 7:**
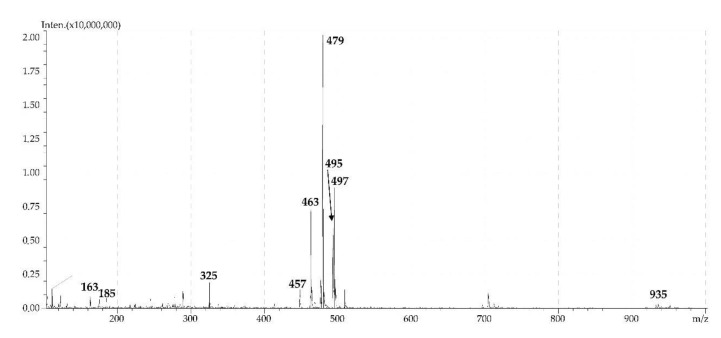
ESI-MS spectrum (positive ionization) of umbelliferone-7-*O*-(6″-apiosyl)-glucoside (6″-apiosylskimmin, **27**).

The dominant highly polar coumarin of the roots of *P. sibiricus* was umbelliferone-7-*O*-(6″-apiosyl)-glucoside or 6″-apiosylskimmin (**27**), identified by comparison with the corresponding reference compound. In the mass spectrum of **27**, a characteristic feature was the high intensity of the peaks of the adduct ions with *m*/*z* 463 [M + Li]^+^, 479 [M + Na]^+^ and 495 [M + K]^+^, as well as the presence of weak signals of the deapiosylated fragment with *m*/*z* 325 [(M + H) – Api]^+^ and deglucosylated fragment (aglycone) with *m*/*z* 163 [(M + H) – Api – Glc]^+^ (Figure 7). Earlier, 6″-apiosylskimmin (**27**) was isolated from the herb *P. sibiricus* [12] and its presence in the roots was established for the first time. Compound **26** was isomeric to **27** and identified as umbelliferone-*O*-desoxyhexosyl-*O*-hexoside.

The three components **28**, **29** and **32** gave a protonated ion with *m/z* 427 in the mass spectrum and were identified as monoglucosides of peucedanol on the basis of the data obtained. Compared to reference compounds, two components were identified as the known Apiaceous coumarins peucedanol-7-*O*-glucoside (**28**) [28] and peucedanol-3′-*O*-glucoside (**28**) (also isolated from *Phlojodicarpus turczaninovii* Sipliv.) [14]. Considering the fact that peucedanol has three hydroxyl groups at C7, C2′ and C3′, isomeric compound **29** is attributed the structure of peucedanol-2′-*O*-glucoside, first discovered in *Seseli montanum* L. [43]. All three compounds are reported here for the first time in *P. sibiricus*.

Finally, 35 coumarins were detected in the roots and herb of *P. sibiricus*, and only seven of them were previously discovered in this plant species. These were umbelliferone (**1**) [8], khellactone (**3**), khellactone 4′-*O*-methyl ester (**4**), hyuganin D (**12**) [9], visnadin (**13**) [6], dihydrosamidin (**15**) [7] and suksdorfin (**16**) [9]. Twenty-eight coumarins (compounds **2**, **5**–**11**, **14**, **17**–**32**, **39**–**41**) were newly discovered components of *P. sibiricus*.

#### 2.2.4. Phenylpropanoids and Flavonoids

Six non-coumarins were detected only in *P. sibiricus* herb extract and were identified by comparison with reference substances such as the phenylpropanoids 1-*O*-caffeoyl-glucose (**33**), 1-*O*-caffeoylquinic acid (**34**), 6-*O*-caffeoyl-glucose (**35**) and 5-*O*-caffeoylquinic acid (**36**), and the two flavonoids diosmetin-7-*O*-glucoside (**37**) and chrysoeriol-7-*O*-glucoside (**38**). Only diosmetin-7-*O*-glucoside (**37**) was described as a *P. sibiricus* flavone [11].

### 2.3. Quantification Assay for Seven Coumarins and One Caffeoylquinic Acid in P. sibiricus Plant Organs and Remedies by Microcolumn HPLC-UV

To carry out a quantitative assessment of the main compounds found in *P. sibiricus*, a chromatographic separation technique using microcolumn chromatography with UV detection (mc-HPLC-UV) was developed. During preliminary studies, chromatographic profiles of extracts from *P. sibiricus* were studied and it was found that the level of the chromatographic signal was sufficient to quantify seven coumarins and one phenylpropanoid. As quantifiable compounds, six esters of khellactone including khellactone 3′,4′-di-*O*-acetyl ester (**9**), khellactone 4′-*O*-angeloyl ester (d-laserpitin, **11**), khellactone 3′-*O*-acetyl-4′-*O*-isobutyroyl ester (hyuganin D, **12**), khellactone 3′-*O*-isovaleroyl-4′-*O*-acetyl ester (dihydrosamidin, **15**), khellactone 3′-*O*-acetyl-4′-*O*-(2-methylbutyroyl) ester (hyuganin C, **17**), khellactone 3′-*O*-glucoside (praeroside II, **31**), as well as umbelliferone-7-*O*-(6′-apiosyl)-glucoside (6′-apiosylskimmin, **27**) and 5-*O*-caffeoylquinic acid (**36**) were selected.

**Figure 8:**
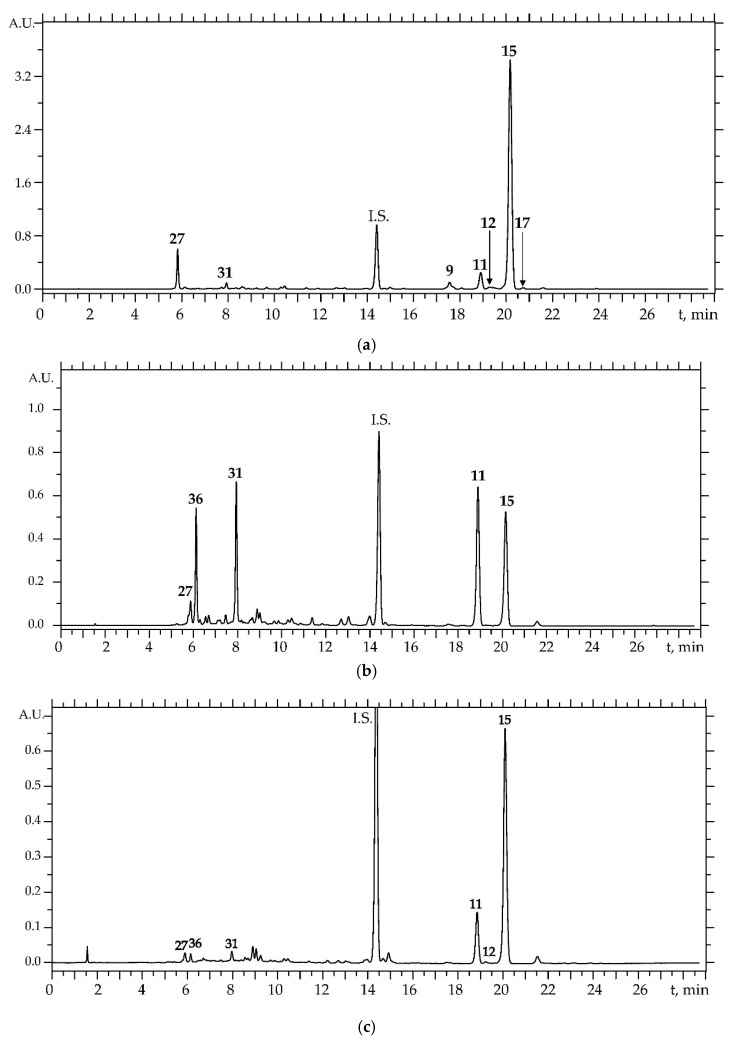
HPLC-UV chromatograms of *P. sibiricus* roots (**a**), herb (**b**) and seeds (**c**). Compounds numbered as **9**—khellactone-3′,4′-di-*O*-acetyl ester, **11**—khellactone 4′-*O*-angeloyl ester (d-laserpitin), **12**—khellactone 3′-*O*-acetyl-4′-*O*-isobutyroyl ester (hyuganin D), **15**—khellactone 3′-*O*-isovaleroyl-4′-*O*-acetyl ester (dihydrosamidin), **17**—khellactone 3′-*O*-acetyl-4′-*O*-(2-methylbutyroyl) ester (hyuganin C), **27**—umbelliferone-7-*O*-(6′-apiosyl)-glucoside (6′-apiosylskimmin), **31**—khellactone-3′-*O*-glucoside (praeroside II), **36**—5-*O*-caffeoylquinic acid. I.S.—internal standard (pimpinellin).

In the process of selecting the optimal conditions for the separation of eight compounds, the effect of various types of columns with reversed-phase sorbent (Kromasil 100-3.5-C_18_, LiChrosorb RP18 5 μm, Nucleosil-3-C_18_, ProntoSIL 120-5-C_18_ AQ, Eurospher 100-5-C_18_), eluent systems (MeCN–H_2_O, MeOH–H_2_O) and additives (HCOOH, CH_3_COOH, CH_3_COONa, CF_3_COOH, HClO_4_, LiClO_4_), column temperatures (25–60 °C), eluent rates (50–600 μL/min) and a mobile phase gradient program were studied. As a result, a method of chromatographic analysis on a ProntoSIL 120-5-C_18_ AQ column with a MeCN/HClO_4_–LiClO_4_/HClO_4_/H_2_O mobile phase was developed, allowing satisfactory separation of the main compounds detected in *P. sibiricus* within 30 min (Figure 8). Furocoumarin pimpinellin (6,7-dimethoxyangelicin), undetected in *P. sibiricus*, was used as an internal standard to improve the quality of method validation criteria. Compounds gave symmetrical peaks with the values of peak asymmetry factors 0.98–1.04 and theoretical plate number more than 35,000 (Table S5). Regression equation analysis demonstrated good linearity for “concentration—chromatographic peak square” dependencies with correlation coefficients of about 0.9999 (Table S6). Limits of detection (LOD) and limits of quantification (LOQ) values were 0.46–0.93 μg/mL and 1.40–2.81 μg/mL, respectively, and linear ranges varied from 2 to 1000 μg/mL. RSD levels of precision, repeatability, stability and recovery parameters were 0.97–2.57%, 1.21–2.59%, 1.52–2.86%, and 97.34–102.64%, respectively (Table S7). In the process of selecting the optimal extraction method suitable both for roots and herb of *P. sibiricus*, it was found that 80% methanol had the highest extraction power (Table S8), and ultrasonic extraction was more effective than microwave-assisted extraction, boiled water bath extraction or room temperature extraction (Table S9).

The developed technique was the first used for the quantitative analysis of the roots, herb and seeds of *P. sibiricus*. Dihydrosamidin (**15**) and 6′-apiosylskimmin (**27**), measuring 80.14 and 7.47 mg/g respectively (Table 3), were the dominant compounds in the roots of *P. sibiricus*. D-laserpitin (**11**), dihydrosamidin (**15**), and praeroside II (**31**) with concentrations 12.51, 10.85 and 10.59 mg/g, respectively, were basic components of *P. sibiricus* herb. Total coumarin content in roots was 98.24 mg/g and was higher than the herb level at 36.16 mg/g. Surprisingly, the seeds were able to concentrate good levels of dihydrosamidin (12.28 mg/g), although not to the same extent as in the roots or herb. In general, it is possible that different organs of *P. sibiricus* may accumulate various components or be capable of organ-specific accumulation of metabolites. Despite the lack of information on the concentration of dihydropyranocoumarins of the khellactone ester group in plant raw materials, it can be noted that some plants also accumulate it, but in considerably lower amounts, as with visnadin (4.5–4.6 mg/g) in *Ammi visnaga* fruits [44] and praeruptorin A (3.68–7.59 mg/g) and praeruptorin B (0.38–2.74 mg/g) in *Peucedanum praeruptorum* roots [45]. In this regard, *P. sibiricus* can be defined as a unique natural concentrator of khellactone esters.

In addition to the quantitative profile of unprocessed plants, we determined the composition of some preparations of *P. sibiricus* roots and herb. Decoctions, the most frequently used *P. sibiricus* preparations, were depleted in the coumarins with total content 0.32 and 0.34 mg/mL, respectively for roots and herb decoction (Table 3). Khellactone esters content was in low or trace level, in contrast to the glycosidic compounds 6′-apiosylskimmin (**27**) and praeroside II (**31**) prevailing in decoctions.

Actually, this was expected because the unpolar nature of khellactone esters needed organic solvents to ease the transition into the preparation (Table S8). That clearly shows quantitative data of the tinctures prepared with 40% ethanol. The sum of coumarins was 5.02 and 2.60 mg/mL, respectively for the roots and herb tinctures. General chromatographic profiles of tinctures were close to those of source plant material. The pharmacological effectiveness of *P. sibiricus* tinctures has already been demonstrated on the model of experimental atherosclerosis of rabbits, while root decoction showed more pronounced anti-cholisterinemic activity than herb decoctions [22]. It is strongly suspected that the high coumarin content in *P. sibiricus* root tincture was the leading cause of high effectiveness. Analysis of oil preparations is another example of mc-HPLC-UV method application. The solution (5%) of the hexane-soluble fraction of *P. sibiricus* root in peach oil was previously recommended as an effective spasmolytic drug in various diseases [46]. A main component of this drug was dihydrosamidin (**15**) with content of 49.96 mg/mL, and the concentrations of other coumarins (for example **9**, **11**, **12**) were no more than 3.00 mg/mL. Recommended daily uptake of dihydrosamidin (150 mg per day) [22] could be satisfied by 3 mL of the root oil solution. Hence, this mc-HPLC-UV method could be used for the quality control of *P. sibiricus* roots and herb in various preparations.

### 2.4. Siberian Populations of P. sibiricus: Comparision of HPLC and Principal Component Analysis Data

The area of distribution of *P. sibiricus* in Siberia is limited to the north by the valley of the Lena River and in the south by Manchuria in Mongolia. The existence of botanical species in various regions of Asia with different climatic conditions may affect their chemical composition. Previously, strong geographical impact was observed for *Angelica archangelica* L. [47], *Crithmum maritimum* L. [48] and some other species able to accumulate various groups of coumarins. To illustrate this fact, we collected and analysed by HPLC (qualitatively and quantitatively) samples of the roots of *P. sibiricus*, as a morphological group with the highest content of coumarins, at the northernmost tip of central Yakutia, in the centre of the Chita region, and in the southern areas of the territory of Buryatia and Manchurian Mongolia. Data on the HPLC profile of two species of *Phlojodicarpus* (*P. villosus* and *P. turczaninovii*) growing inside the specified territory were also obtained.

**Figure 9:**
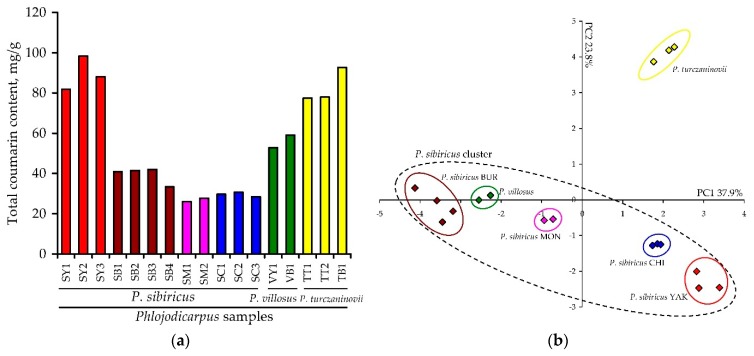
(**a**) Total coumarin content in roots of *Phlojodicarpus* samples (S—*P. sibiricus*, V—*P. villosus*, T—*P. turczaninovii*) from various regions (B—Buryatia, C—Chita, M—Mongolia, Y—Yakutia). (**b**) Results of principal component analysis (PCA) used the content of 18 coumarins in the roots of seventeen *Phlojodicarpus* samples (BUR—Buryatia, CHI—Chita, MON—Mongolia, YAK—Yakutia).

As a result, a chromatographic analysis of 17 *Phlojodicarpus* samples allowed us to establish the HPLC profile for each studied group of samples (Figure S4). The dominance of dihydrosamidin (**15**) and its high content (67.08–80.14 mg/g) were the distinguishing features of northern samples of *P. sibiricus* from Yakutia (Table S10). None of the other investigated groups was similar to this group. 6′-apiosyl-skimmin (**27**) prevailed in the southern samples from Buryatia and Mongolia, and additional unidentified compounds **i**–**viii** were found; these were not detected in Yakut samples, and they form a group of lipophilic coumarins with a high retention time (t_R_ 20.5–24.0 min). The concentration of **15** in the roots from Buryatia was 0.22–0.59 mg/g, and in samples from Mongolia it was 1.27–1.90 mg/g. D-Laserpitin (**11**) dominated in the roots of *P. sibiricus* from Chita (19.69–21.52 mg/g), and the content of **15** was 3.37–4.49 mg/g. The concentration of lipophilic coumarins **i**–**viii** was low but detectable, unlike samples from Yakutia. The total content of coumarins in the roots of *P. sibiricus* from Yakutia was 81.82–98.24 mg/g; in the roots from Buryatia, Chita and Mongolia it was 33.39–41.93, 28.38–30.63 and 25.92–27.61 mg/g, respectively (Figure 9a).

The composition and quantitative data on the content of coumarins in samples from four populations of *P. sibiricus* indicate the existence of at least three chemotypes of this species. The first type is northern (Yakutia), which is characterised by the highest content of coumarins and is a source of dihydrosamidin (**15**). The southern type (Buryatia, Mongolia) is geographically opposite and contains a different set of coumarins (additional lipophilic coumarins) and very few of those in **15**. The *P. sibiricus* population from Chita belongs to a transition type with an average content the same as **15**, traces of additional lipophilic coumarins and the prevalence of D-laserpitin (**11**).

*P. villosus* and *P. turczaninovii* species are morphologically close to *P. sibiricus* and are also found in the indicated collection areas, but they differ in their HPLC profiles (Figure S4); therefore, there is no reason to assume any confusion when identifying *P. sibiricus* species. In particular, *P. villosus* is close to *P. sibiricus* due to the predominance of 6′-apiosylskimmin (**27**) and the presence of additional lipophilic coumarins; the southern *P. sibiricus* population is relatively abundant in D-laserpitin (**11**) and dihydrosamidin (**15**) as a transitional population of *P. sibiricus*. In general, the HPLC profile of *P. villosus* is different. Moreover, there are additional compounds **ix**–**xi** in the roots of *P. turczaninovii* which were not found in *P. sibiricus* or *P. villosus*, indicating its undeniable chemical differences from these species. As for the total concentration of coumarins, *P. villosus* is characterised by intermediate values of 52.75–58.99 mg/g, while *P. turczaninovii* is close to *P. sibiricus* (77.42–92.63 mg/g) (Figure 9a).

Considering the HPLC data obtained using principal component analysis (PCA), there exist two zones with the highest concentration of variables (Figure 9b). The total scores for the PCA reached 61.7% of variables PC1 (37.9%) and PC2 (23.8%). The data on *P. sibiricus* populations are distributed over a wide range of PC1 axis values, forming a large cluster designated as the *P. sibiricus* cluster. In the negative region of PC1 there are densely grouped southern sets of *P. sibiricus* from Buryatia and Mongolia characterised by a low content of compounds **9**, **11**, **15**, **17** and a high content of **i**–**viii**. In the area of positive PC1 values, there is a transitional population of *P. sibiricus* from the Chita region with an average content of **9**, **11**, **15**, **17**, and the northern population from Yakutia with the highest concentration of **15**. Inside the *P. sibiricus* cluster there is a group of data belonging to *P. villosus* which indicates the proximity of this species to *P. sibiricus*. The group of variables for *P. turczaninovii* is a considerable distance from the main cluster and indicates the marked differences in its chemical composition from that of *P. sibiricus* and *P. villosus*. Thus, the joint analysis of chromatographic information and mathematical modeling data clearly indicates the existence of a geographic variation of coumarin accumulation in the roots of *P. sibiricus*. It is possible that in the future we could select the most appropriate population of *P. sibiricus* for specific purposes (botanical, genomic, pharmacological), one that meets the necessary requirements of the chemical profile.

### 2.5. Dihydrosamidin as an Antiobesity Principle of P. sibiricus Roots

To identify the most active antiobesity agent in the hexane fraction of *P. sibiricus* roots, we studied three khellactone derivatives: khellactone 3′-*O*-isovaleroyl-4′-*O*-acetyl ester (dihydro-samidin, **15**), khellactone 4′-*O*-angeloyl ester (d-laserpitin, **11**) and khellactone 3′-*O*-glucoside (praeroside II, **31**), as well as non-khellactone compounds such as umbelliferone-7-*O*-(6′-apiosyl)-glucoside (6′-apiosylskimmin, **27**) and diosmetin-7-*O*-glucoside (**37**). Differentiated 3T3-L1 adipocytes were treated with pure compounds for 2–6 days. Total triacylglycerol content in the cells was lower in dihydrosamidin-treated cells using both 10 μg/mL (22.6% from control level) and 20 μg/mL (19.8% from control level) formulations (Table 4). Khellactone 4′-*O*-angeloyl ester was less active, with inhibition levels of 64.4% (10 μg/mL) and 59.5% (20 μg/mL).

At the concentrations used (10, 20 μg/mL) khellactone 3′-*O*-glucoside, umbelliferone-7-*O*-(6′-apiosyl)-glucoside and diosmetin-7-*O*-glucoside were inactive. Therefore, dihydrosamidin was identified as the most active antiobesity agent in *P. sibiricus* roots.

The influence of the nature of the substituents of the *O*-esters of khellactone on their biological activity was previously considered in the examples of spasmolytic [49], vasodilatory [50], cAMP-phosphodiesterase (cAMP-PDE) inhibitory [51], anti-HIV [52], anticancer [53], P-glycoprotein modulating [54] and anti-lipid accumulative activities [3]. Summarising the known data, the unsubstituted khellactone, as well as its glucosides, was always less effective or ineffective [51].

The presence of monoacetyl or diacetyl substituents slightly increased the activity of dihydropyranocoumarins, but not as in the case of five-carbon acyloxy groups [49,50,51]. The esters of khellactone with isovaleric, senecionic, angelic and 2-methylbutyric acids demonstrated high biological activity, while the 3′,4′-*cis*-configuration was more effective than *trans*-isomers [3]. Detection of high antiobesity effectiveness of dihydrosamidin confirms early data about structure-activity relationships among khellactone derivatives. It is interesting that its isomer visnadin or khellactone 3′-*O*-(2-methylbutyroyl)-4′-*O*-acetyl ester was previously assumed as the most effective antiobesity component of *Peucedanum japonicum* [3]. Effectiveness of monosubstituted khellactone 4′-*O*-angeloyl ester or D-laserpitin (**11**) was intermediate and most likely caused by the absence of the neighbouring acetyl group in the 3′-*O*-position or less potency of the angeloyl substituent compared with isovaleroyl group.

## 3. Materials and Methods

### 3.1. Plant Materials and Chemicals

The samples of roots, herb and seeds of *P. sibiricus* were collected in various regions: SY1—Bulunsky District, Republic of Sakha; 26.VIII.2016; 53°59′26″ N, 108°89′17″ E, voucher specimens No. CRo/an-03/23-25/0716; SY2—Aldansky District, Republic of Sakha; 29.VII.2016; 59°0′34.042″ N, 130°51′39.2432″ E, voucher specimens No. CRo/an-03/24-29/0716; SY3—Amginsky District, Republic of Sakha; 01.VIII.2016; 61°2′13.2440″ N, 133°12′33.2227″ E, voucher speciens No. CRo/an-03/23-28/0816; SB1—Barguzin District, Republic of Buryatia; 26.VIII.2016; 53°59′26″ N, 108°89′17″ E, voucher specimens No. CRo/an-03/23-25/0716; SB2—Tunkinsky District, Republic of Buryatia; 23.VII.2016; 51°38′42.2916″ N, 102°6′30.5228″ E, voucher specimen No. CRo/an-03/28-21/0716; SB3—Kyakhtinsky District, Republic of Buryatia; 30.VII.2016; 50°21′57.7828″ N, 106°24′25.3839″ E, voucher specimens No. CRo/an-03/21-25/0716; SB4—Okinsky District, Republic of Buryatia; 02.VIII.2016; 52°36′0.46181″ N, 99°49′7.2748″ E, voucher specimens No. CRo/an-03/20-26/0816; SM1—Arkhangai Province, Mongolia; 03.VIII.2016; 47°51′53.1863″ N, 101°0′11.3434″ E, voucher specimens No. CRo/an-03/19-25/0816; SM2—Dornod Province, Mongolia; 04.VIII.2016; 47°40′38.2012″ N, 115°13′3.7758″ E, voucher specimens No. CRo/an-03/19-23/0816; SC1—Agin-Buryat Autonomous Okrug, Zabaykalsky Krai; 28.VII.2016; 51°6′40.6155″ N, 114°31′23.5236″ E, voucher specimens No. CRo/an-03/21-22/0716; SC2—Shilkinsky District, Zabaykalsky Krai; 29.VII.2016; 51°50′57.6699″ N, 116°3′15.9137″ E, voucher specimens No. CRo/an-03/23-24/0716; SC3—Borzinsky District, Zabaykalsky Krai; 30.VII.2016; 50°23′57.5769″ N, 116°37′17.7228″ E, voucher specimens No. CRo/an-03/26-29/0716. The samples of roots of *P. villosus* were collected in various regions: VY1—Mirninsky District, Republic of Sakha; 07.VIII.2016; 62°33′3.4485″ N, 114°0′7.3334″ E, voucher specimens No. CRo/an-03/23-29/0816; VB1—Kizhinginsky District, Republic of Buryatia; 19.VII.2016; 51°50′37.8179″ N, 109°56′43.8254″ E, voucher specimens No. CRo/an-03/20-29/0716. The samples of roots of *P. turczaninovii* were collected in various regions: TT1—Bay-Tayginsky District, Tuva Republic; 12.VIII.2016; 51°0′49.5168″ N, 90°12′56.8021″ E, voucher specimens No. CRo/an-03/20-21/0816; TT2—Ovyursky District, Tuva Republic; 14.VIII.2016; 50°43′51.2905″ N, 92°3′56.0103″ E, voucher specimens No. CRo/an-03/23-28/0816; TB1—Zaigrayevsky District, Republic of Buryatia; 31.VII.2016; 51°55′18.0023″ N, 108°3′4.8690″ E, voucher specimens No. CRo/an-03/26-280716. The species were authenticated by Prof. T.A. Aseeva (IGEB SB RAS, Ulan-Ude). Plant material was dried and powdered before analysis.

The chemicals were purchased from ChemFaces (Wuhan, Hubei, PRC)—khellactone (Cat. No. CFN96394, ≥98%), khellactone 4′-*O*-methyl ester (Cat. No. CFN89413, ≥98%), khellactone 4′-*O*-angeloyl ester (d-laserpitin; Cat. No. CFN92710, ≥98%), khellactone 3′-*O*-acetyl-4′-*O*-isobutyroyl ester (hyuganin D; Cat. No. CFN89402, ≥98%), khellactone 3′-*O*-acetyl-4′-*O*-angeloyl ester (pteryxin; Cat. No. CFN92559, ≥98%), khellactone 3′,4′-di-*O*-angeloyl ester (praeruptorin D; Cat. No. CFN98142, ≥98%), khellactone-3′-*O*-glucoside (praeroside II; Cat. No. CFN96695, ≥98%), pimpinellin (Cat. No. CFN99402, ≥98%), umbelliferone-7-*O*-(6′-apiosyl)-glucoside (6′-apiosylskimmin; Cat. No. CFN90311, ≥98%), peucedanol-7-*O*-glucoside (Cat. No. CFN89412, ≥98%), peucedanol-3′-*O*-glucoside (Cat. No. CFN89411, ≥98%), 1-*O*-caffeoylquinic acid (Cat. No. CFN99121, ≥98%), chrysoeriol-7-*O*-glucoside (thermopsoside; Cat. No. CFN93021, ≥98%); Sigma-Aldrich (St. Louis, MO, USA)—acetonitrile for HPLC (Cat. No 34851, ≥99.9%), aluminum chloride hydrate (Cat. No. 229393); ammonium oxalate monohydrate (Cat. No. 221716, ≥99%), khellactone 3′-*O*-(2-methylbutyroyl)-4′-*O*-acetyl ester (visnadin; Cat. No. SMB00087, ≥95%), lithium perchlorate (Cat. No. 431567, ≥99%), 3-methyl-1-phenyl-2-pyrazoline-5-one (Cat. No. M70800, ≥99%), oxalic acid (Cat. No. 194131, ≥98%), perchloric acid 70% (Cat. No. 311421, ≥99%), phenol (Cat. No. P1037, ≥99%); sulfuric acid (Cat. No. 258105, ≥95%); trifluoroacetic acid (Cat. No. T6508, ≥99%), umbelliferone (Cat. No. H24003, ≥99%), 5-*O*-caffeoylquinic acid (Cat. No. 94419, ≥98%); VILAR Corp. (Moscow, Russia)—khellactone 4′-*O*-acetyl ester (Cat. No. V602, ≥95%), khellactone 3′-*O*-isovaleroyl-4′-*O*-acetyl ester (dihydrosamidin; Cat. No. V127, ≥98%), khellactone 3′-O-acetyl-4′-O-(2-methylbutyroyl) ester (hyuganin C; Cat. No. VC0475, ≥94%), khellactone-3′,4′-di-O-acetyl ester (Cat. No. VC0814, ≥95%), khellactone 3′,4′-di-O-senecioyl ester (Cat. No. V1025, ≥95%); Extrasynthese (Lyon, France)—chlorogenic acid (Cat. No. 4991 S, ≥99%), bergapten (Cat. No. 0552 S, ≥99%), diosmetin-7-*O*-glucoside (Cat. No. 1376 S, ≥98%); MedKoo Biosciences, Inc. (Morrisville, NC, USA)—khellactone 3′-*O*-acetyl-4′-*O*-isovaleroyl ester (suksdorfin; Cat. No. 461845, ≥98%). 1-*O*-Caffeoylglucose and 6-*O*-caffeoylglucose were isolated previously from *Calendula officinalis* [55] and *Filipendula ulmaria* [56].

Samples of tinctures from *Phlojodicarpus sibiricus* roots (batch no. 120616) and *Phlojodicarpus sibiricus* herb (batch no. 241117) were purchased from Arura Corp. (Ulan-Ude, Russia); *Phlojodicarpus sibiricus* roots oil solution (also known as Dimidin) was purchased from VILAR Corp (batch no. 201018). Equipment used for UV-Vis spectrophotometry was a SF-2000 UV-Vis-spectrophotometer (OKB Specter, St. Petersburg, Russia).

### 3.2. Chemical Composition Analysis

Essential oil content was determined gravimetrically after hydrodistillation in a Clevenger apparatus [57]. The total flavonoid content was estimated as rutin equivalents by a spectrophotometric procedure after 5% AlCl_3_ addition [58]. The total caffeoylquinic acid content was determined by the colorimetric Arnow method using 3-*O*-caffeoylquinic acid as the standard [59]. Total content of carbohydrate polymers (water-soluble polysaccharides and pectic substances) was determined with spectrophotometric phenol–sulphuric acid method [60]. Essential oil component was analysed by GC/MS method on a 6890N gas chromatograph (Agilent Technologies, city, state abbrev if USA, country) coupled to a Agilent Technologies 5973 N mass selective/quadrupole detector using a fused capillary column HP-5MS (30 m × 0.25 mm, film thickness 0.50 μm, 5% diphenyl- and 95% dimethylpolysiloxane stationary phase) [61]. The monosaccharide composition of polysaccharides was determined after acidic hydrolysis with trifluoroacetic acid (TFA) following by 1-phenyl-3-methyl-5-pyrazolone (PMP) labeling and microcolumn HPLC with ultraviolet detection separation (HPLC-UV) of PMP-labeled hydrolyzates [57,62].

### 3.3. Total Coumarin Content Spectrophotometric Assay

Accurately weighted powdered plant material (1 g) was mixed with 80% methanol (50 mL) in a conical flask (150 mL) and agitated in an ultrasonic bath for 30 min. The resulted solution was filtered into a volumetric flask (100 mL) and extraction was repeated again in the same conditions. The final volume in volumetric flask was reached to 100 mL with 80% methanol (solution A). One millilitre of soln. A was placed into volumetric flask (25 mL), and the final volume was reached to 25 mL with 80% methanol (solution B). The optical density of soln. B was measured at 328 nm using 80% methanol as blank solution. Dihydrosamidin was used as the reference compound. To prepare the stock solutions, dihydrosamidin (10 mg) was accurately weighed and dissolved in 80% methanol in volumetric flask (10 mL). The standard calibration curve was generated using six data points, including concentration points 3.125, 6.250, 12.500, 25.000, 50.000, 100.000 µg/mL. The calibration curves were created by plotting the optical density vs. the concentration levels. All the analyses were carried out in triplicate and the data were expressed as mean value ± standard deviation (SD).

### 3.4. Inhibition of Triacylglycerol Accumulation in Differentiated 3T3-L1 Adipocytes

3T3-L1 preadipocytes were purchased from Sigma (Cat. No. 86052701) and maintained in Dulbecco′s modified Eagle′s medium (DMEM, Sigma, Cat. No. D5030) with 10% bovine calf serum (BCS, Sigma, Cat. No. 12133C) and avoided complete confluence before initiating differentiation [3]. For adipogenesis, preadipocytes were cultured in 24-well plates (10^4^ cells per well) and differentiation was induced for 48 h by DMEM supplemented by 10% BCS, 0.05 mM 3-isobutyl-1-methylxanthine (Sigma, Cat. No. I5879), 0.25 μM dexamethasone (Sigma, Cat. No. D4902), 10 μg/mL insulin (Sigma, Cat. No. I3536), and *P. sibiricus* extracts and fractions (50 μg/mL, soln. in DMSO) or pure compounds (10, 20 μg/mL, soln. in DMSO) [63]. The control group was cultured in the basic maintenance medium without *P. sibiricus* extracts, fractions or pure compounds. At the end of 6 day, the cells were washed with 0.1 M phosphate buffer solution (pH 7.4), lysed with 1% Triton™ X-100 (BioUltra, Cat. No. 93443), and total lipids were extracted accordingly Bligh-Dyer method [64]. Triacylglycerol content was determined spectrophotometrically (540 nm) using Sigma kit (Cat. No. TR0100) according to manufacturer’s instruction.

### 3.5. Total Extract, Fractions and Decoction Preparation

For preparation of the total extract the powdered sample of *P. sibiricus* (roots, herb or seeds; 100 g) was transferred in a conical glass flask (2 L). Methanol solution (80%, 1 L) was added with stirring and mixture was sonicated for 60 min at 50 °C under 100 W ultrasound power at a frequency of 35 kHz. The extraction was repeated three times and resulted extracts were filtered through a cellulose filter, combined and evaporated *in vacuo* until dryness. The total extracts were stored at 4 °C until further chemical composition analysis and bioactivity assays. The yields of total extracts of *P. sibiricus* organs were 28.14 g, 34.22 g and 9.93 g, respectively.

For preparation of the total fractions of *P. sibiricus* roots, 25 g of dry extract was suspended in 200 mL of water and extracted by hexane (100 mL) three times. Hexane and water phases were separately evaporated *in vacuo* until dryness. The yields of hexane and water fractions were 9.75 and 15.20 g, respectively. Essential oil was isolated after hydrodistillation of dry roots in Clevenger apparatus [57] with yield 2.6% from dry plant weight. Polysaccharide fractions of water soluble polysaccharides and pectic substances were extracted with water and 0.5% oxalic acid—0.5% ammonium oxalate mixture, respectively, using the methods described previously [62]. The yields of the water soluble polysaccharides and pectic substances were 2.58% and 1.92% form dry weight of *P. sibiricus* roots.

For preparation of decoction, an accurately weighted *P. sibiricus* sample (roots or herb; 1 g) was placed in conical flasks. Then 100 mL of distilled water was added and the samples were heated on heater plate and boiled for 10 min. The mixture was left to stand at room temperature for 15 min, and then filtered under reduced pressure.

### 3.6. HPLC-DAD-ESI-QQQ-MS/MS Profiling Condition

Reversed-phase high-performance liquid chromatography with diode array detection and electrospray ionization mass spectrometry (RP-HPLC-DAD-ESI-TQ-MS/MS) procedure was used for phenolic compounds profiling. Experiments were performed on an LCMS 8050 liquid chromatograph coupled with diode-array-detector and triple-quadrupole electrospray ionization detector (Shimadzu, Columbia, MD, USA), using a GLC Mastro C18 column (150 × 2.1 mm, Ø 3 μm; Shimadzu, Kyoto, Japan), column temperature was 35°C. Eluent A was water and eluent B was acetonitrile. The injection volume was 1 μL, and elution flow was 100 μL/min. Gradient program: 0–10 min 10–20% B, 10–30 min 20–100% B, 30–32 min 100% B. The DAD acquisitions were performed in the range of 200–600 nm and chromatograms were integrated at 330 nm. For ESI-MS, the parameters were set as follows: temperature levels of ESI interface, desolvation line and heat block were 300 °C, 250 °C and 400 °C, respectively; the flow levels of nebulizing gas (N_2_), heating gas (air) and collision-induced dissociation gas (Ar) were 3 L/min, 10 L/min and 0.3 mL/min, respectively. The capillary voltage was kept at +3 kV (coumarins) in positive mode and at −4.0 kV (phenylpropanoids and flavonoids) in negative mode. ESI-MS spectra were recorded by scanning in the range of *m*/*z* 100–1900.

### 3.7. Microcolumn HPLC-UV (mc-HPLC-UV) Quantification Condition

Quantification of phenolic compounds was realized in mc-HPLC-UV experiments using microcolumn HPLC apparatus. Experiments were performed on an microcolumn chromatograph Econova MiLiChrom A-02 (Novosibirsk, Russia), using a ProntoSIL-120-5-C18 AQ column (1 × 50 mm, Ø 1 μm; Metrohm AG; Herisau, Switzerland), column temperature was 30 °C. Eluent A was 0.2 M LiClO_4_ in 0.01 M HClO_4_ and eluent B was 0.01 M HClO_4_ in acetonitrile. The injection volume was 1 μL, and elution flow was 150 μL/min. Gradient program: 0.0–26.6 min 5–100% B, 26.6–28.6 min 100% B. The chromatograms were recorded at 330 nm.

To prepare the stock solutions of reference compounds, 9 mg khellactone-3′,4′-di-*O*-acetyl ester, khellactone 4′-*O*-angeloyl ester (d-laserpitin), khellactone 3′-*O*-acetyl-4′-*O*-isobutyroyl ester (hyuganin D), khellactone 3′-*O*-isovaleroyl-4′-*O*-acetyl ester (dihydrosamidin), khellactone 3′-*O*-acetyl-4′-*O*-(2-methylbutyroyl) ester (hyuganin C), umbelliferone-7-*O*-(6′-apiosyl)-glucoside (6′-apiosylskimmin), khellactone-3′-*O*-glucoside (praeroside II), and 5-*O*-caffeoylquinic acid were accurately weighed and individually dissolved in methanol in volumetric flasks (1 mL). The external standard calibration curve was generated using eight data points, covering the concentration ranges 1–1000 µg/mL. The calibration curves were created by plotting the peak area vs. the concentration levels. Pimpinellin (t_R_ 14.35 min) was used as the internal standards and was dissolved separately in methanol at concentration 9 mg/mL. All the analyses were carried out in triplicate and the data were expressed as mean value ± standard deviation (SD). Quantification of unidentified components **i**–**xi** was done using dihydrosamidin as reference compound. For preparation of sample solution, an accurately weighted powdered plant (40 mg) was placed in an Eppendorf tube, 1 mL of 80% methanol was added, and the mixture was weighted. Then the sample was extracted in an ultrasonic bath for 30 min at 50 °C. After cooling, the tube weight was reduced to initial sign, and the resultant extract was filtered through a 0.22-μm PTFE syringe filter before injection into the HPLC system for analysis.

### 3.8. Method Validation

For validation of the analytical method, we used the procedure described previously [65]. The linearity of the method was studied by injecting five known concentrations of the standard compounds in the defined range. Results from each analysis were averaged and subjected to regression analysis. Limits of detection (LOD) and quantification (LOQ) were determined using the following equations: LOD = (3.3 × *S*_YX_)/*a*; LOQ = (10 × *S*_YX_)/*a*, where *S*_YX_ is a standard deviation of the response (Y intercept) and *a* is a slope of calibration curve. The precision of the analytical method was evaluated by intra-day, inter-day, and repeatability test. Intra-day assay was determined by assaying the mixture solution containing 8 standards (50 µg/mL) during the same day (five injections), and inter-day assay was analyzed using the same concentration for intra-day precision on four different days (interval of 1 day) in the same laboratory. The repeatability test of the sample was performed on sevenfold experiments of the mixture solution containing 8 standards (100 µg/mL). The stability test was performed with one sample solution, which was stored at room temperature and analyzed at 0, 2, 4, 8, 12, 24 and 48 h. For analysis of recovery data, the appropriate amounts of the powdered sample of 8 standards were weighted and spiked with a known amount of each reference compound and then analyzed. Each sample was analyzed in five times.

### 3.9. Statistical and Multivariative Analysis

Statistical analyses were performed using a one-way analysis of variance (ANOVA), and the significance of the mean difference was determined by Duncan’s multiple range test. Differences at *p* < 0.05 were considered statistically significant. The results are presented as mean values ± SD (standard deviations). Advanced Grapher 2.2 (Alentum Software Inc., Ramat-Gan, Israel) was used to perform linear regression analysis and to generate graphs. Principal component analysis (PCA) based on a data matrix (18 markers × 17 samples) was performed using Graphs 2.0 utility for Microsoft Excel (Komi NTc URO RAN, Syktyvkar, Russia) to generate an overview for groups clustering.

## 4. Conclusions

It is obvious that for manifestation of the greatest antiobesity activity of dihydropyrano- coumarins with the khellactone skeleton, they must contain two substituents at C-3′ and C-4′, one of which is an acetyl group, and the second a five-carbon acyloxy group. In conclusion, this study has increased our knowledge of the structure-activity relationships for the antiobesity effect of khellactone derivative coumarins. Furthermore, as an important result of our research to prove the ethnopharmacological data of *P. sibiricus* application, dihydrosamidin as an active natural coumarin has been shown to be an antiobesity agent in plant material of *P. sibiricus*. Additional studies are needed to deepen our knowledge about mechanisms of antiobesity activity of dihydrosamidin and *P. sibiricus* plant remedies.

## Figures and Tables

**Table 1:** Total content of essential oil, coumarins, flavonoids, caffeoylquinic acids (CQAs), water-soluble polysaccharides (WSPS) and pectic substances (PS) in *Phlojodicarpus sibiricus* organs and triacylglycerol (TG) content in 3T3-L1 adipocytes after incubation with *P. sibiricus* extracts ^1^.

Extract Type	Essential Oil, mg/g ^a,2^	Coumarins, mg/g ^a,2^	Flavonoids, mg/g ^a,2^	CQAs, mg/g ^a,2^	WSPS, mg/g ^a,2^	PS, mg/g ^a,2^	TG, μg/mg Protein ^b,3,4^
*P. sibiricus* roots	7.52 ± 0.31	108.94 ± 1.96	Traces	Traces	2.58 ± 0.05	2.27 ± 0.04	526.4 ± 20.5 *
*P. sibiricus* herb	2.11 ± 0.08	56.28 ± 1.06	Traces	18.62 ± 0.46	3.74 ± 0.07	2.61 ± 0.05	755.1 ± 35.1 *
*P. sibiricus* seeds	Traces	5.62 ± 0.10	Traces	Traces	2.11 ± 0.04	1.90 ± 0.03	809.7 ± 32.3

^1^ Averages ± standard deviation were obtained from three (^a^) or four (^b^) different experiments. ^2^ Dry plant weight. ^3^ *P. sibiricus* extracts concentration 50 μg/mL. TG content in control group—812.8 ± 25.1 μg/mg protein. TG content in positive control group (5-*O*-caffeoylquinic acid, 10 μg/mL)—286.2 ± 11.45 * μg/mg protein. ^4^ Values with asterisk (*) indicate statistically significant differences with the control groups at *p* < 0.05 by one-way ANOVA.

**Table 2:** Identified compounds in *P. sibiricus* root and herb extracts.

No	T_R_, min	UV Pattern ^1^	Found in		Compound	Molecular Formula (m.w.)	ESI-MS Data, *m*/*z* (Intensity, %) ^4^
			Roots	Herb			
1	15.03	A	+		Umbelliferone ^2^	C_9_H_6_O_3_ (162.1)	185 (100) [M + Na]^+^, 163 (8) [M + H]^+^
2	15.67	A	+		Bergapten ^2^	C_12_H_8_O_4_ (216.2)	257 (5) [(M + Na) + H_2_O]^+^, 255 (47) [M + K]^+^, 239 (100) [M + Na]^+^, 217 (2) [M + H]^+^
3	16.18	A	+		Khellactone ^2^	C_14_H_14_O_5_ (262.3)	301 (9) [M + K]^+^, 285 (100) [M + Na]^+^, 269 (60) [M + Li]^+^
4	17.35	A	+	+	Khellactone 4′-*O*-methyl ester ^2^	C_15_H_16_O_5_ (276.3)	315 (33) [M + K]^+^, 299 (100) [M + Na]^+^, 283 (18) [M + Li]^+^
5	17.71	A	+		Khellactone 4′-*O*-isovaleroyl/2-methylbutyroyl ester ^3^	C_19_H_22_O_6_ (346.4)	715 (<1) [2M + Na]^+^, 387 (5) [(M + Na) + H_2_O]^+^, 385 (15) [M + K]^+^, 369 (100) [M + Na]^+^, 365 (6) [(M + H) + H_2_O]^+^, 353 (12) [M + Li]^+^, 347 (2) [M + H]^+^, 267 (6) [(M + Na) – C_5_H_10_O_2_]^+^, 245 (5) [(M + H) – C_5_H_10_O_2_]^+^, 227 (<1) [(M + H) – C_5_H_10_O_2_ – H_2_O]^+^, 199 (<1) [(M + H) – C_5_H_10_O_2_ – H_2_O – CO]^+^
6	18.24	A	+	+	Khellactone 4′-*O*-acetyl ester ^2^	C_16_H_16_O_6_ (304.4)	343 (7) [M + K]^+^, 327 (100) [M + Na]^+^, 323 (<1) [(M + H) + H_2_O]^+^, 311 (47) [M + Li]^+^, 305 (<1) [M + H]^+^, 267 (5) [(M + Na) – C_2_H_4_O_2_]^+^, 245 (<1) [(M + H) – C_2_H_4_O_2_]^+^
7	18.63	A	+		Khellactone 4′-*O*-isovaleroyl/2-methylbutyroyl ester ^3^	C_19_H_22_O_6_ (346.4)	715 (<1) [2M + Na]^+^, 387 (6) [(M + Na) + H_2_O]^+^, 385 (27) [M + K]^+^, 369 (100) [M + Na]^+^, 365 (5) [(M + H) + H_2_O]^+^, 353 (61) [M + Li]^+^, 347 (<1) [M + H]^+^, 267 (9) [(M + Na) – C_5_H_10_O_2_]^+^, 245 (1) [(M + H) – C_5_H_10_O_2_]^+^, 227 (<1) [(M + H) – C_5_H_10_O_2_ – H_2_O]^+^, 199 (<1) [(M + H) – C_5_H_10_O_2_ – H_2_O – CO]^+^
8	19.87	A	+		Lomatin *O*-isovaleroyl/2-methylbutyroyl ester ^3^	C_19_H_22_O_5_ (330.4)	387 (7) [(M + K)+H_2_O]^+^, 371 (1) [(M + Na)+H_2_O]^+^, 369 (63) [M + K]^+^, 355 (9) [(M + Li)+H_2_O]^+^, 353 (100) [M + Na]^+^, 349 (23) [(M + H) + H_2_O]^+^, 337 (47) [M + Li]^+^, 331 (7) [M + H]^+^, 269 (20) [(M + Na) – C_5_H_8_O]^+^, 247 (24) [(M + H) – C_5_H_8_O]^+^
9	22.43	A	+		Khellactone 3′,4′-di-*O*-acetyl ester ^2^	C_19_H_22_O_6_ (346.4)	387 (14) [(M + Na)+H_2_O]^+^, 385 (28) [M + K]^+^, 369 (100) [M + Na]^+^, 365 (24) [(M + H) + H_2_O]^+^, 353 (48) [M + Li]^+^, 347 (<1) [M + H]^+^, 309 (16) [(M + Na) – C_2_H_4_O_2_]^+^, 287 (12) [(M + H) – C_2_H_4_O_2_]^+^, 367 (52) [(M + Na) – C_2_H_4_O_2_ – C_2_H_2_O]^+^, 245 (16) [(M + H) – C_2_H_4_O_2_ – C_2_H_2_O]^+^, 227 (14) [(M + H) – C_2_H_4_O_2_ – C_2_H_2_O – H_2_O]^+^, 199 (5) [(M + H) – C_2_H_4_O_2_ – C_2_H_2_O – H_2_O – CO]^+^
10	23.20	A	+		Khellactone 4′-*O*-isobutyroyl ester ^3^	C_18_H_20_O_6_ (332.4)	371 (21) [M + K]^+^, 355 (100) [M + Na]^+^, 351 (2) [(M + H) + H_2_O]^+^, 339 (12) [M + Li]^+^, 245 (2) [(M + H) – C_4_H_8_O_2_]^+^
11	24.89	A	+		Khellactone 4′-*O*-angeloyl ester (d-laserpitin) ^2^	C_19_H_20_O_6_ (344.4)	711 (<1) [2M + Na]^+^, 385 (11) [(M + Na) + H_2_O]^+^, 383 (22) [M + K]^+^, 367 (100) [M + Na]^+^, 363 (7) [(M + H) + H_2_O]^+^, 351 (11) [M + Li]^+^, 345 (<1) [M + H]^+^, 267 (5) [(M + Na) – C_5_H_8_O_2_]^+^, 245 (2) [(M + H) – C_5_H_8_O_2_]^+^
12	25.14	A	+	+	Khellactone 3′-*O*-acetyl-4′-*O*-isobutyroyl ester (hyuganin D) ^2^	C_20_H_22_O_7_ (374.4)	771 (<1) [2M + Na]^+^, 415 (11) [(M + Na) + H_2_O]^+^, 413 (14) [M + K]^+^, 397 (100) [M + Na]^+^, 393 (16) [(M + H) + H_2_O]^+^, 381 (10) [M + Li]^+^, 287 (11) [(M + H) – C_4_H_8_O_2_]^+^, 245 (<1) [(M + H) – C_4_H_8_O_2_ – C_2_H_2_O]^+^, 227 (<1) [(M + H) – C_4_H_8_O_2_ – C_2_H_2_O – H_2_O]^+^, 199 (<1) [(M + H) – C_4_H_8_O_2_ – C_2_H_2_O – H_2_O – CO]^+^
13	25.27	A	+		Khellactone 3′-*O*-(2-methylbutyroyl)-4′-*O*-acetyl ester (visnadin) ^2^	C_21_H_24_O_7_ (388.4)	815 (5) [2M + K]^+^, 799 (100) [2M + Na]^+^, 783 (54) [2M + Li]^+^, 429 (15) [(M + Na)+H_2_O]^+^, 427 (63) [M + K]^+^, 411 (99) [M + Na]^+^, 407 (6) [(M + H) + H_2_O]^+^, 395 (25) [M + Li]^+^, 389 (<1) [M + H]^+^, 351 (<1) [(M + Na) – C_2_H_4_O_2_]^+^, 329 (57) [(M + H) – C_2_H_4_O_2_]^+^, 245 (<1) [(M + H) – C_2_H_4_O_2_ – C_5_H_8_O]^+^, 227 (<1) [(M + H) – C_2_H_4_O_2_ – C_5_H_8_O – H_2_O]^+^, 199 (<1) [(M + H) – C_2_H_4_O_2_ – C_5_H_8_O – H_2_O – CO]^+^
14	25.64	A	+		Khellactone 3′-*O*-acetyl-4′-*O*-angeloyl ester (pteryxin) ^2^	C_21_H_22_O_7_ (386.4)	811 (<1) [2M + K]^+^, 795 (37) [2M + Na]^+^, 779 (11) [2M + Li]^+^, 427 (23) [(M + Na) + H_2_O]^+^, 425 (51) [M + K]^+^, 409 (100) [M + Na]^+^, 405 (11) [(M + H) + H_2_O]^+^, 393 (50) [M + Li]^+^, 387 (<1) [M + H]^+^, 309 (<1) [(M + Na) – C_5_H_8_O_2_]^+^, 287 (18) [(M + H) – C_5_H_8_O_2_]^+^, 245 (<1) [(M + H) – C_5_H_8_O_2_ – C_5_H_6_O]^+^, 227 (<1) [(M + H) – C_5_H_8_O_2_ – C_5_H_6_O – H_2_O]^+^, 199 (<1) [(M + H) – C_5_H_8_O_2_ – C_5_H_6_O – H_2_O – CO]^+^
15	26.01	A	+	+	Khellactone 3′-*O*-isovaleroyl-4′-*O*-acetyl ester (dihydrosamidin) ^2^	C_21_H_24_O_7_ (388.4)	815 (<1) [2M + K]^+^, 799 (83) [2M + Na]^+^, 783 (40) [2M + Li]^+^, 429 (23) [(M + Na) + H_2_O]^+^, 427 (55) [M + K]^+^, 411 (100) [M + Na]^+^, 407 (12) [(M + H) + H_2_O]^+^, 406 (55) [M + H_2_O]^+^, 395 (42) [M + Li]^+^, 388 (<1) [M + H]^+^, 329 (85) [(M + H) – C_2_H_4_O_2_]^+^, 245 (5) [(M + H) – C_2_H_4_O_2_ – C_5_H_8_O]^+^, 227 (<1) [(M + H) – C_2_H_4_O_2_ – C_5_H_8_O – H_2_O]^+^, 199 (<1) [(M + H) – C_2_H_4_O_2_ – C_5_H_8_O – H_2_O – CO]^+^
16	26.05	A	+		Khellactone 3′-*O*-acetyl-4′-*O*-isovaleroyl ester (suksdorfin) ^2^	C_21_H_24_O_7_ (388.4)	799 (16) [2M + Na]^+^, 783 (2) [2M + Li]^+^, 429 (10) [(M + Na) + H_2_O]^+^, 427 (16) [M + K]^+^, 411 (100) [M + Na]^+^, 407 (15) [(M + H) + H_2_O]^+^, 395 (21) [M + Li]^+^, 309 (<1) [(M + Na) – C_5_H_10_O_2_]^+^, 287 (25) [(M + H) – C_5_H_10_O_2_]^+^, 245 (5) [(M + H) – C_5_H_10_O_2_ – C_2_H_2_O]^+^, 227 (<1) [(M + H) – C_5_H_10_O_2_ – C_2_H_2_O – H_2_O]^+^, 199 (<1) [(M + H) – C_5_H_10_O_2_ – C_2_H_2_O – H_2_O – CO]^+^
17	27.23	A	+		Khellactone 3′-*O*-acetyl-4′-*O*-(2-methylbutyroyl) ester (hyuganin C) ^2^	C_21_H_24_O_7_ (388.4)	799 (22) [2M + Na]^+^, 783 (15) [2M + Li]^+^, 429 (20) [(M + Na) + H_2_O]^+^, 427 (27) [M + K]^+^, 411 (100) [M + Na]^+^, 407 (7) [(M + H) + H_2_O]^+^, 395 (24) [M + Li]^+^, 309 (<1) [(M + Na) – C_5_H_10_O_2_]^+^, 287 (12) [(M + H) – C_5_H_10_O_2_]^+^, 245 (10) [(M + H) – C_5_H_10_O_2_ – C_2_H_2_O]^+^, 227 (<1) [(M + H) – C_5_H_10_O_2_ – C_2_H_2_O – H_2_O]^+^, 199 (<1) [(M + H) – C_5_H_10_O_2_ – C_2_H_2_O – H_2_O – CO]^+^
18	27.63	A	+		Khellactone 3′,4′-di-*O*-senecioyl ester ^2^	C_24_H_26_O_7_ (426.5)	875 (11) [2M + Na]^+^, 860 (7) [2M + Li]^+^, 467 (10) [(M + Na) + H_2_O]^+^, 465 (4) [M + K]^+^, 449 (100) [M + Na]^+^, 445 (2) [(M + H) + H_2_O]^+^, 433 (15) [M + Li]^+^, 427 (<1) [M + H]^+^, 349 (<1) [(M + Na) – C_5_H_8_O_2_]^+^, 327 (10) [(M + H) – C_5_H_8_O_2_]^+^, 245 (<1) [(M + H) – C_5_H_8_O_2_ – C_5_H_6_O]^+^
19	27.97	A	+		Khellactone 3′,4′-di-*O*-angeloyl ester (praeruptorin D) ^2^	C_24_H_26_O_7_ (426.5)	875 (5) [2M + Na]^+^, 860 (2) [2M + Li]^+^, 467 (16) [(M + Na) + H_2_O]^+^, 465 (17) [M + K]^+^, 449 (100) [M + Na]^+^, 445 (6) [(M + H) + H_2_O]^+^, 433 (23) [M + Li]^+^, 427 (<1) [M + H]^+^, 349 (<1) [(M + Na) – C_5_H_8_O_2_]^+^, 327 (5) [(M + H) – C_5_H_8_O_2_]^+^, 245 (<1) [(M +H) – C_5_H_8_O_2_ – C_5_H_6_O]^+^
20	28.00	A	+		Khellactone 3′-*O*-isovaleroyl/2-methylbutyroyl-4′-*O*- senecioyl/angeloyl ester ^3^	C_24_H_28_O_7_ (428.5)	895 (<1) [2M + K]^+^, 879 (5) [2M + Na]^+^, 863 (<1) [2M + Li]^+^, 485 (6) [(M + K) + H_2_O]^+^, 469 (22) [(M + Na) + H_2_O]^+^, 467 (15) [M + K]^+^, 453 (12) [(M + Li) + H_2_O]^+^, 451 (100) [M + Na]^+^, 446 (22) [(M + H) + H_2_O]^+^, 435 (34) [M + Li]^+^, 429 (<1) [M + H]^+^, 329 (21) [(M + H) – C_5_H_8_O_2_]^+^, 245 (24) [(M + H) – C_5_H_8_O_2_ – C_5_H_8_O]^+^, 227 (5) [(M + H) – C_5_H_8_O_2_ – C_5_H_8_O – H_2_O]^+^, 199 (<1) [(M + H) – C_5_H_8_O_2_ – C_5_H_8_O – H_2_O – CO]^+^
21	28.19	A	+		Khellactone 3′-*O*-isovaleroyl/2-methylbutyroyl-4′-*O*- isobuturoyl ester ^3^	C_23_H_28_O_7_ (416.5)	871 (<1) [2M + K]^+^, 855 (<1) [2M + Na]^+^, 473 (7) [(M + K) + H_2_O]^+^, 457 (14) [(M + Na) + H_2_O]^+^, 455 (13) [M + K]^+^, 441 (20) [(M + Li) + H_2_O]^+^, 439 (100) [M + Na]^+^, 435 (9) [(M + H) + H_2_O]^+^, 423 (20) [M + Li]^+^, 417 (<1) [M + H]^+^, 329 (28) [(M + H) – C_4_H_8_O_2_]^+^, 245 (<1) [(M + H) – C_4_H_8_O_2_ – C_5_H_8_O]^+^, 227 (12) [(M + H) – C_4_H_8_O_2_ – C_5_H_8_O – H_2_O]^+^
22	28.51	A	+		Khellactone 3′-*O*-senecioyl/angeloyl-4′-*O*-isovaleroyl/ 2-methylbutyroyl ester ^3^	C_24_H_28_O_7_ (428.5)	895 (<1) [2M + K]^+^, 879 (11) [2M + Na]^+^, 863 (<1) [2M + Li]^+^, 485 (9) [(M + K) + H_2_O]^+^, 469 (23) [(M + Na) + H_2_O]^+^, 467 (18) [M + K]^+^, 453 (27) [(M + Li) + H_2_O]^+^, 451 (100) [M + Na]^+^, 446 (24) [(M + H) + H_2_O]^+^, 435 (32) [M + Li]^+^, 429 (<1) [M + H]^+^, 327 (26) [(M + H) – C_5_H_10_O_2_]^+^, 245 (26) [(M + H) – C_5_H_10_O_2_ – C_5_H_6_O]^+^, 227 (10) [(M + H) – C_5_H_10_O_2_ – C_5_H_6_O – H_2_O]^+^, 199 (<1) [(M + H) – C_5_H_10_O_2_ – C_5_H_6_O – H_2_O – CO]^+^
23	28.59	A	+		Khellactone 3′,4′-di-*O*-isovaleroyl/2-methylbutyroyl ester / khellactone isovaleroyl-2-methylbutyroyl ester ^3^	C_24_H_30_O_7_ (430.5)	899 (<1) [2M + K]^+^, 883 (15) [2M + Na]^+^, 867 (2) [2M + Li]^+^, 487 (5) [(M + K) + H_2_O]^+^, 471 (14) [(M + Na) + H_2_O]^+^, 469 (22) [M + K]^+^, 455 (31) [(M + Li) + H_2_O]^+^, 453 (100) [M + Na]^+^, 449 (12) [(M + H) + H_2_O]^+^, 437 (35) [M + Li]^+^, 431 (<1) [M + H]^+^, 329 (22) [(M + H) – C_5_H_10_O_2_]^+^, 245 (4) [(M + H) – C_5_H_10_O_2_ – C_5_H_8_O]^+^, 227 (10) [(M + H) – C_5_H_10_O_2_ – C_5_H_8_O – H_2_O]^+^
24	28.63	A	+		Khellactone 3′,4′-di-*O*-isoburyoyl ester ^3^	C_22_H_26_O_7_ (402.5)	459 (2) [(M + K) + H_2_O]^+^, 443 (8) [(M + Na) + H_2_O]^+^, 441 (8) [M + K]^+^, 427 (12) [(M + Li) + H_2_O]^+^, 425 (100) [M + Na]^+^, 409 (63) [M + Li]^+^, 403 (14) [M + H]^+^, 315 (11) [(M + H) – C_4_H_8_O_2_]^+^, 245 (5) [(M + H) – C_4_H_8_O_2_ – C_4_H_6_O]^+^, 227 (7) [(M + H) – C_4_H_8_O_2_ – C_4_H_6_O – H_2_O]^+^
25	29.02	A	+		Khellactone 3′,4′-di-*O*-isovaleroyl/2-methylbutyroyl ester / khellactone isovaleroyl-2-methylbutyroyl ester ^3^	C_24_H_30_O_7_ (430.5)	899 (<1) [2M + K]^+^, 883 (16) [2M + Na]^+^, 867 (7) [2M + Li]^+^, 487 (7) [(M + K) + H_2_O]^+^, 471 (11) [(M + Na) + H_2_O]^+^, 469 (24) [M + K]^+^, 455 (35) [(M + Li) + H_2_O]^+^, 453 (100) [M + Na]^+^, 449 (10) [(M + H) + H_2_O]^+^, 437 (37) [M + Li]^+^, 431 (<1) [M + H]^+^, 329 (28) [(M + H) – C_5_H_10_O_2_]^+^, 245 (2) [(M + H) – C_5_H_10_O_2_ – C_5_H_8_O]^+^, 227 (12) [(M + H) – C_5_H_10_O_2_ – C_5_H_8_O – H_2_O]^+^
26	8.32	A	+	+	Umbelliferone-*O*-desoxyhexosyl-*O*-hexoside ^3^	C_20_H_24_O_12_ (456.4)	495 (21) [M + K]^+^, 479 (100) [M + Na]^+^, 475 (7) [(M + H) + H_2_O]^+^, 463 (25) [M + Li]^+^, 325 (9) [(M + H) – dHex]^+^, 185 (2) [(M + Na) – dHex – Hex]^+^, 163 (1) [(M +H) – dHex – Hex]^+^
27	8.57	A	+	+	Umbelliferone-7-*O*-(6″-apiosyl)-glucoside (6″-apiosylskimmin) ^2^	C_20_H_24_O_12_ (456.4)	935 (<1) [2M + Na]^+^, 497 (35) [(M + Na) + H_2_O]^+^, 495 (39) [M + K]^+^, 479 (100) [M + Na]^+^, 475 (10) [(M + H) + H_2_O]^+^, 463 (38) [M + Li]^+^, 347 (<1) [(M + Na) – Api]^+^, 325 (8) [(M + H) – Api]^+^, 185 (1) [(M + Na) – Api – Glc]^+^, 163 (2) [(M + H) – Api – Glc]^+^
28	10.49	A	+		Peucedanol-7-*O*-glucoside ^2^	C_20_H_26_O_10_ (426.4)	465 (27) [M + K]^+^, 449 (100) [M + Na]^+^, 433 (14) [M + Li]^+^, 427 (1) [M + H]^+^, 287 (2) [(M + Na) – Glc]^+^, 265 (10) [(M + H) – Glc]^+^
29	12.53	A	+		Peucedanol-2′-*O*-glucoside (tentative) ^3^	C_20_H_26_O_10_ (426.4)	465 (31) [M + K]^+^, 449 (100) [M + Na]^+^, 433 (12) [M + Li]^+^, 427 (2) [M + H]^+^, 287 (7) [(M + Na) – Glc]^+^, 265 (12) [(M + H) – Glc]^+^
30	12.57	A	+	+	Khellactone-4′-*O*-glucoside (tentative) ^3^	C_20_H_24_O_10_ (424.4)	463 (33) [M + K]^+^, 447 (100) [M + Na]^+^, 431 (14) [M + Li]^+^, 285 (<1) [(M + Na) – Glc]^+^, 263 (6) [(M + H) – Glc]^+^
31	13.81	A	+	+	Khellactone-3′-*O*-glucoside (praeroside II) ^2^	C_20_H_24_O_10_ (424.4)	887 (<1) [2M + K]^+^, 871 (6) [2M + Na]^+^, 463 (69) [M + K]^+^, 447 (100) [M + Na]^+^, 431 (<1) [M + Li]^+^, 425 (5) [M + H]^+^, 285 (<1) [(M + Na) – Glc]^+^, 263 (3) [(M + H) – Glc]^+^
32	14.15	A	+	+	Peucedanol-3′-*O*-glucoside ^2^	C_20_H_26_O_10_ (426.4)	465 (24) [M + K]^+^, 449 (100) [M + Na]^+^, 433 (26) [M + Li]^+^, 431 (10) [(M + Na) – H_2_O]^+^, 427 (2) [M + H]^+^, 305 (5) [(M + Na + H_2_O) – Glc]^+^, 287 (6) [(M + Na) – Glc]^+^, 283 (8) [(M + H + H_2_O) – Glc]^+^, 265 (8) [(M + H) – Glc]^+^
33	1.04	B		+	1-*O*-Caffeoyl-glucose ^2^	C_15_H_18_O_9_ (342.3)	341 (100) [M − H]^−^, 179 (11) [(M − H) – Glc]^-^
34	1.29	B		+	1-*O*-Caffeoylquinic acid ^2^	C_16_H_18_O_9_ (354.3)	353 (100) [M − H]^−^, 191 (52) [(M − H) – Caf]^−^, 179 (44) [Caf − H]^−^
35	3.61	B		+	6-*O*-Caffeoyl-glucose ^2^	C_15_H_18_O_9_ (342.3)	341 (100) [M − H]^−^, 179 (22) [(M − H) – Glc]^−^
36	5.82	B		+	5-*O*-Caffeoylquinic acid ^2^	C_16_H_18_O_9_ (354.3)	353 (100) [M − H]^−^, 191 (61) [(M − H) – Caf]^−^, 179 (37) [Caf − H]^−^
37	10.43	C		+	Diosmetin-7-*O*-glucoside ^2^	C_22_H_22_O_11_ (462.4)	461 (100) [M − H]^−^, 299 (14) [(M − H) – Glc]^−^, 285 (4) [(M − H) – Glc – 14]^−^
38	10.71	C		+	Chrysoeriol-7-*O*-glucoside ^2^	C_22_H_22_O_11_ (462.4)	461 (100) [M − H]^−^, 299 (25) [(M − H) – Glc]^−^, 285 (7) [(M − H) – Glc – 14]^−^
39	15.51	A		+	Khellactone *O*-isobutyryl ester (tentative) ^3^	C_18_H_20_O_6_ (332.4)	371 (38) [M + K]^+^, 355 (100) [M + Na]^+^
40	16.12	A		+	Khellactone *O*-hexoside ^3^	C_20_H_24_O_10_ (424.4)	871 (6) [2M + Na]^+^, 463 (40) [M + K]^+^, 447 (100) [M + Na]^+^, 263 (2) [(M + H) – Hex]^+^
41	20.75	A		+	Khellactone 3′-*O*-methyl ester (tentative) ^3^	C_15_H_16_O_5_ (276.3)	315 (47) [M + K]^+^, 299 (100) [M + Na]^+^, 263 (2) [(M + H) – CH_2_]^+^

^1^ For UV spectra pattern see Figure S2. ^2^ Compound identification was based on comparison with reference standard. ^3^ Compound identification was based on interpretation of UV and MS spectral data and comparison with literature data. ^4^ Functional group abbreviations: Api—apiosyl, Caf—caffeoyl, dHex—desoxyhexosyl, Glc—glucosyl, Hex—hexosyl. “+”—presence of compound.

**Table 3:** Content of 8 compounds and sum of coumarins (ΣCou) in unprocessed *P. sibiricus* plant material and *P. sibiricus* preparations ^a^.

Compound ^b^	Roots ^c^	Herb ^c^	Seeds ^c^	Root Decoction ^d^	Herb Decoction ^d^	Root Tincture ^d^	Herb Tincture ^d^	Root Oil Solution ^d^
**9**	2.52 ± 0.11	Tr.	Tr.	N.d.	N.d.	0.15 ± 0.00	Tr.	0.65 ± 0.01
**11**	4.70 ± 0.09	12.51 ± 0.24	2.14 ± 0.04	N.d.	N.d.	0.21 ± 0.00	0.64 ± 0.01	1.44 ± 0.02
**12**	1.49 ± 0.02	Tr.	0.10 ± 0.00	N.d.	N.d.	0.07 ± 0.00	Tr.	0.86 ± 0.02
**15**	80.14 ± 1.44	10.85 ± 0.21	12.28 ± 0.025	0.03 ± 0.00	Tr.	3.84 ± 0.08	0.87 ± 0.02	49.96 ± 0.98
**17**	0.53 ± 0.02	Tr.	Tr.	Tr.	N.d.	0.05 ± 0.00	Tr.	Tr.
**27**	7.47 ± 0.14	2.21 ± 0.04	0.40 ± 0.01	0.25 ± 0.00	0.04 ± 0.00	0.59 ± 0.01	0.18 ± 0.00	N.d.
**31**	1.39 ± 0.03	10.59 ± 0.22	0.46 ± 0.01	0.04 ± 0.00	0.30 ± 0.01	0.11 ± 0.00	0.91 ± 0.00	N.d.
**36**	Tr.	4.60 ± 0.09	0.14 ± 0.00	Tr.	0.12 ± 0.00	Tr.	0.39 ± 0.01	N.d.
ΣCou	98.24	36.16	15.42	0.32	0.34	5.02	2.60	52.91

^a^ Median ± S.D. ^b^ Compounds: **9**—khellactone-3′,4′-di-*O*-acetyl ester, **11**—khellactone 4′-*O*-angeloyl ester (d-laserpitin), **12**—khellactone 3′-*O*-acetyl-4′-*O*-isobutyroyl ester (hyuganin D), **15**—khellactone 3′-*O*-isovaleroyl-4′-*O*-acetyl ester (dihydrosamidin), **17**—khellactone 3′-*O*-acetyl-4′-*O*-(2-methylbutyroyl) ester (hyuganin C), **27**—umbelliferone-7-*O*-(6′-apiosyl)-glucoside (6′-apiosylskimmin), **31**—khellactone-3′-*O*-glucoside (praeroside II), **36**—5-*O*-caffeoylquinic acid. ^c^ mg/g of dry plant weight. ^d^ mg/mL. N.d.—not detected (<LOD). Tr.—traces (<LOQ).

**Table 4:** Triacylglycerol (TG) content in 3T3-L1 adipocytes after incubation with pure compounds ^a^.

Compound	Concentration, μg/mL	TG, μg/mg Protein ^b^
Khellactone 4′-*O*-angeloyl ester (d-laserpitin, 11)	10	523.6 ± 20.9 *
	20	483.6 ± 20.7 *
Khellactone 3′-*O*-isovaleroyl-4′-*O*-acetyl ester (dihydrosamidin, 15)	10	184.2 ± 7.3 *
	20	161.7 ± 6.9 *
Umbelliferone-7-*O*-(6′-apiosyl)-glucoside (6′-apiosylskimmin, 27)	10	806.1 ± 32.2
	20	804.5 ± 40.0
Khellactone-3′-*O*-glucoside (praeroside II, 31)	10	792.0 ± 34.5
	20	763.4 ± 30.5
Diosmetin-7-*O*-glucoside (37)	10	802.6 ± 33.7
	20	801.5 ± 33.0
5-*O*-Caffeoylquinic acid (reference compound)	10	286.2 ± 11.4 *
Control (water)	-	812.8 ± 25.1

^a^ Averages ± standard deviation were obtained from four different experiments. ^b^ Values with asterisk (*) indicate statistically significant differences with the control groups at *p* < 0.05 by one-way ANOVA.

## Supplementary Materials

The following are available online. Table S1. Coumarins found in *Phlojodicarpus* genus; Table S2. Triacylglycerol content in 3T3-L1 preadipocytes after incubation with *P. sibiricus* root fractions; Table S3. Essential oil composition of *P. sibiricus* roots; Table S4. Monosaccharide composition of crude water-soluble polysaccharide fraction and crude pectic substances fraction from *P. sibiricus* roots; Table S5. Retention times, peak asymmetry factors, and theoretical plane number for 8 compounds; Table S6. Regression equations, correlation coefficients, standard deviation, limits of detection, limits of quantification and linear ranges for 8 compounds; Table S7. Intra- and inter-day precision, repeatability, stability and recovery for 8 compounds; Table S8. Content of 8 compounds and total coumarin content in water-methanol mixtures media after extraction of *Phlojodicarpus sibiricus* roots and herb; Table S9. Content of 8 compounds and sum of coumarins in water-methanol (20:80, *v*/*v*) media after extraction of *P. sibiricus* roots and herb by various type of extraction; Table S10. Content of compounds **9**, **11**, **12**, **15**, **17**, **27**, **31**, **i**–**xi** in roots of 17 *Phlojodicarpus* samples; Figure S1. Correlation graphs between total coumarin content in 12 samples of *Phlojodicarpus sibiricus* roots and their influence on tiacylglycerol content in 3T3-L1 preadipocytes; Figure S2. Structures of reference standards used in present work; Figure S3. Types of UV spectral patterns; Figure S4. mc-HPLC-UV chromatograms of *P. sibiricus* roots extracts prepared with various solvents; Figure S5. mc-HPLC-UV chromatograms of *P. sibiricus* herb extracts prepared with various solvents; Figure S6. mc-HPLC-UV chromatograms of *Phlojodicarpus* root extracts from various regions.
