# Supplementary material for: Khellactone Derivatives and Other Phenolics of Phlojodicarpus sibiricus (Apiaceae): HPLC-DAD-ESI-QQQ-MS/MS and HPLC-UV Profile, and Antiobesity Potential of Dihydrosamidin
Source: Molecules. 2019 Jun 19;24(12):2286. doi: 10.3390/molecules24122286 (PMC6630902; doi:10.3390/molecules24122286)
Supplement: Supplementary file 1 [file molecules-24-02286-s001.pdf]

molecules

Supplementary materials

Khellactone Derivatives and Other Phenolics of *Phlojodicarpus sibiricus* (Apiaceae): HPLC-DAD-ESI-QQQ-MS/MS and HPLC-UV Profile, and Antiobesity Potential of Dihydrosamidin

Daniil N. Olennikov [1,*], Innokentii A. Fedorov [2], Nina I. Kashchenko [1], Nadezhda K. Chirikova [3], and Cecile Vennos [4]

1. Institute of General and Experimental Biology, Siberian Division, Russian Academy of Science, 6 Sakh'yanovoy Street, Ulan-Ude 670047, Russia; ninkk@mail.ru
2. Institute for Biological Problems of Cryolithozone, Siberian Division, Russian Academy of Science, 41 Lenina Street, Yakutsk, 677000, Russia; fedorovia1958@mail.ru
3. Department of Biochemistry and Biotechnology, North-Eastern Federal University, 58 Belinsky Street, Yakutsk 677027, Russia; hofnung@mail.ru
4. Regulatory and Medical Scientific Affairs, Padma AG, 1 Underfeldstrasse, Hinwil CH-8340, Switzerland; vennos_c@mail.ru

* Correspondence: olennikovdn@mail.ru; Tel.: +7-902-160-0627

Abstract: With obesity, the consumption of phenolic-enriched food additives as a part of traditional nutrition avoids the negative implications of eating high-calorie products. This study investigated the new herbal food additive, *Phlojodicarpus sibiricus* roots and herb, ubiquitously used in Siberia as a spice. Chromatographic techniques such as HPLC-DAD-ESI-QQQ-MS/MS and microcolumn HPLC-UV were the basic instruments for component profiling and quantification, and antiobesity potential was investigated using a differentiated 3T3-L1 adipocytes assay. We found that the roots and herb of *P. sibiricus* were high-coumarin-containing additives inhibiting triacylglycerol accumulation in 3T3-L1 preadipocytes. Forty-one phenolics were detected in *P. sibiricus* extracts, and 35 were coumarins, including 27 khellactone derivatives present as esters and glucosides. Total coumarin content varied from 36.16 mg/g of herb to 98.24 mg/g of roots, and from 0.32 mg/mL to 52.91 mg/mL in *P. sibiricus* preparations. Moreover, Siberian populations of *P. sibiricus* were characterised by a different HPLC-based coumarin profile. The most pronounced inhibiting effect on triacylglycerol accumulation in 3T3-L1 preadipocytes was shown for dihydrosamidin (khellactone 3′-*O*-isovaleroyl-4′-*O*-acetyl ester), which was more active than other khellactone esters and glucosides. The results demonstrated that if used as a food additive *Phlojodicarpus sibiricus* could be a source of bioactive coumarins of the khellactone group with high antiobesity potential.

Keywords: *Phlojodicarpus sibiricus*; khellactone esters; dihydrosamidin; HPLC-MS; antiobesity activity; 3T3-L1 adipocytes.

List of Supplementary Content

Table S1. Coumarins found in *Phlojodicarpus* genus.
Table S2. Triacylglycerol content in 3T3-L1 preadipocytes after incubation with *P. sibiricus* root fractions.
Table S3. Essential oil composition of *P. sibiricus* roots.
Table S4. Monosaccharide composition of crude water-soluble polysaccharide fraction and crude pectic substances fraction from *P. sibiricus* roots

Table S5. Retention times, peak asymmetry factors, and theoretical plane number for 8 compounds.
Table S6. Regression equations, correlation coefficients, standard deviation, limits of detection, limits of quantification and linear ranges for 8 compounds.
Table S7. Intra- and inter-day precision, repeatability, stability and recovery for 8 compounds.
Table S8. Content of 8 compounds and total coumarin content in water-methanol mixtures media after extraction of *Phlojodicarpus sibiricus* roots and herb.
Table S9. Content of 8 compounds and sum of coumarins in water-methanol (20:80, *v*/*v*) media after extraction of *P. sibiricus* roots and herb by various type of extraction.
Table S10. Content of compounds **9**, **11**, **12**, **15**, **17**, **27**, **31**, **i-xi** in roots of 17 *Phlojodicarpus* samples.
Figure S1. Correlation graphs between total coumarin content in 12 samples of *Phlojodicarpus sibiricus* roots and their influence on tiacylglycerol content in 3T3-L1 preadipocytes (μg/mg protein).
Figure S2. Structures of reference standards used in present work.
Figure S3. Types of UV spectral patterns.
Figure S4. mc-HPLC-UV chromatograms of *P. sibiricus* roots extracts prepared with various solvents.
Figure S5. mc-HPLC-UV chromatograms of *P. sibiricus* herb extracts prepared with various solvents.
Figure S6. mc-HPLC-UV chromatograms of *Phlojodicarpus* root extracts from various regions.

Table S1. Coumarins found in *Phlojodicarpus* genus.

**Compound**	***P. sibiricus***		***P. turczaninovii***		***P. villosus***	
	**Roots**	**Herb**	**Roots**	**Herb**	**Roots**	**Herb**
Simple coumarins						
Umbelliferone	[8]			[13]		
Umbelliferone 7-*O*-(6′-Api*f*)-Glc*p* (6′-apiosyl-skimmin)		[12]				[11]
Peucedanol 3-*O*-Glc				[14]		
Phlojodicarpin		[10]				
Isophlojodicarpin		[10]				
7-Hydroxy-8-(2',3'-dihydroxy-3'-methylbutyl)-coumarin 7-O-Glc*p*		[11]				
Scopoletin	[8]			[13]		
Furocoumarins						
Isoimperatorin		[12]				
Marmesin				[14]		
Angelicin			[13]			
Columbianetin (zosimol)			[13]	[13]		
Columbianetin 10-*O*-Sen (libanorin)			[13]	[14]		
Columbianetin 10-*O*-Ang (columbianadin, zosimin)			[13]	[13]		
Oroselone				[13]		
Oroselol			[13]	[13]		
8,9-Dihydrooroselol (vaginidiol)			[13]			
8,9-Dihydrooroselol 8-*O*-iVal			[13]			
8,9-Dihydrooroselol 8-*O*-mBu			[13]	[13]		
8,9-Dihydrooroselol 8-*O*-iVal-9-*O*-mBu			[13]			
8,9-Dihydrooroselol 9-*O*-Ac-10-*O*-Sen (peucenidin)			[13]	[14]		
8,9-Dihydrooroselol 9-*O*-Ang-10-*O*-Ac (libanotin, edultin)			[13]	[13]		
Pyranocoumarins						
Decursinol				[14]		[15]
Decursinol 3-*O*-Sen (decursin)						[15]
Decursinol 3-*O*-Ang (agasyllin)						[15]
Lomatin 3′-*O*-Sen (buchtormin, nuttallin)				[14]		
Dihydrolomatin						
Khellactone 4'-*O*-Me		[16]				

Khellactone 3′-*O*-mBu-4′-*O*-Ac (visnadin)	[6]		[6]
Khellactone 3′-*O*-iVal-4′-*O*-Ac (dihydrosamidin)	[7]		[7]
Khellactone 3′-*O*-Ac-4′-*O*-iVal (suksdorfin)		[16]	
Khellactone 3'-*O*-Ac-4'-*O*-mBu		[16]	

Ac – acetyl, Ang – angeloyl, Api*f* – apiosylfuranose, Glc*p* – glucosylpyranose, iVal – isovaleroyl, mBu – 2-methylbutanoyl, Me – methyl, Sen – senecioyl.

Table S2. Triacylglycerol (TG) content in 3T3-L1 adipocytes after incubation with *P. sibiricus* root fractions [a, b]

**Fraction**	**TG, μg/mg protein** [c]
Hexane fraction	316.2 ± 12.8*
Water fraction	627.1 ± 25.1*
Essential oil	783.7 ± 35.2
Water-soluble polysaccharide	815.3 ± 35.6
Pectic substances	809.6 ± 38.0
5-*O*-Caffeoylquinic acid (reference compound)	286.2 ± 11.4*
Control (water)	812.8 ± 25.1

[a] Averages ± standard deviation were obtained from four different experiments. [b] Concentration used 50 μg/mL. [c] Values with asterisk (*) indicate statistically significant differences with the control groups at $p < 0.05$ by one-way ANOVA.

Table S3. Essential oil composition (percentage of total component content) of *P. sibiricus* roots.

**Compound**	**RI**	**MI** [a]	**%**
α-Pinene	932	i, ii, iii	1.5
Sabinene	973	i, ii, iii	40.3
β-Pinene	975	i, ii, iii	3.2
β-Myrcene	991	i, ii, iii	0.2
Δ-Carene	1011	i, ii, iii	2.6
p-Cymene	1024	i, ii, iii	1.5
β-Phellandrene	1027	i, ii, iii	0.2
Limonene	1029	i, ii, iii	30.4
1,8-Cineol	1031	i, ii, iii	0.1
γ-Terpinene	1058	i, ii, iii	0.1
Terpinolene	1089	i, ii, iii	1.9
Linalool	1100	i, ii, iii	0.5
Terpinene-4-ol	1177	i, ii, iii	5.7
Thymol	1292	i, ii, iii	2.7
Carvacrol	1302	i, ii, iii	0.9
α-Copaene	1377	i, ii	0.1
β-Elemene	1392	i, ii, iii	1.2
β-Caryophyllene	1420	i, ii, iii	0.1
Aromadendrene	1441	i, ii, iii	1.2
α-Humulene	1456	i, ii, iii	0.1
β-Selinene	1488	i, ii	1.9
α-Selinene	1497	i, ii	3.3
δ-Cadinene	1527	i, ii, iii	0.1
Caryophyllene oxide	1587	i, ii, iii	0.1
Total			99.9

[a] Methods of identification: i—retention index, ii—mass spectrum, iii—co-injection with authentic sample.

Table S4. Monosaccharide composition of crude water-soluble polysaccharide fraction (WSPS) and crude pectic substances fraction (PS) from *P. sibiricus* roots, mol%.

Monosaccharide	WSPS	PS
Arabinose	1.5	5.1
Fucose	Traces	Traces
Galactose	25.8	7.6
Glucose	59.4	5.1
Mannose	1.4	Traces
Rhamnose	1.9	15.4
Xylose	Traces	Traces
Galacturonic acid	8.3	46.2
Glucuronic acid	1.6	20.5

Table S5. Retention times (t_R), peak asymmetry factors (AsF), and theoretical plate number (N) for 8 compounds and internal standard (I.S.).

Compound	t_R, min	AsF	N
9	15.53	1.02	61256 ± 1102
11	18.86	0.99	75544 ± 1208
12	19.32	1.04	83427 ± 1668
15	20.18	1.01	105894 ± 1482
17	20.76	0.98	127610 ± 2041
27	5.78	0.97	36866 ± 737
31	7.85	1.02	40075 ± 761
36	6.22	1.03	38325 ± 804
I.S.	14.35	1.01	73778 ± 1475

[a] Compounds: **9** – khellactone-3′,4′-di-*O*-acetyl ester, **11** – khellactone 4′-*O*-angeloyl ester (d-laserpitin), **12** – khellactone 3′-*O*-acetyl-4′-*O*-isobutyroyl ester (hyuganin D), **15** – khellactone 3′-*O*-isovaleroyl-4′-*O*-acetyl ester (dihydrosamidin), **17** – khellactone 3′-*O*-acetyl-4′-*O*-(2-methylbutyroyl) ester (hyuganin C), **27** – umbelliferone-7-*O*-(6′-apiosyl)-glucoside (6′-apiosylskimmin), **31** – khellactone-3′-*O*-glucoside (praeroside II), **36** – 5-*O*-caffeoylquinic acid, I.S. – internal standard (pimpinellin).

Table S6. Regression equations, correlation coefficients (r^2), standard deviation (S_{YX}), limits of detection (LOD), limits of quantification (LOQ) and linear ranges for 8 compounds.

Compound	Regression equation	r^2	S_{YX}	LOQ (μg/mL)	LOD (μg/mL)	Linear range (μg/mL)
9	$y = 0.039 \cdot x - 0.007$	0.9999	$8.07 \cdot 10^{-3}$	0.68	2.05	2.5–1000.0
11	$y = 0.040 \cdot x - 0.001$	0.9999	$1.04 \cdot 10^{-2}$	0.83	2.50	3.0–1000.0
12	$y = 0.037 \cdot x - 0.008$	0.9999	$6.18 \cdot 10^{-3}$	0.53	1.62	2.0–1000.0
15	$y = 0.033 \cdot x - 0.010$	0.9999	$8.18 \cdot 10^{-3}$	0.80	2.42	2.5–1000.0
17	$y = 0.032 \cdot x - 0.007$	0.9999	$9.16 \cdot 10^{-3}$	0.93	2.81	3.0–1000.0
27	$y = 0.032 \cdot x - 0.003$	0.9999	$7.49 \cdot 10^{-3}$	0.72	2.18	2.5–1000.0
31	$y = 0.030 \cdot x - 0.010$	0.9999	$8.24 \cdot 10^{-3}$	0.88	2.67	3.0–1000.0
36	$y = 0.057 \cdot x - 0.001$	0.9999	$8.59 \cdot 10^{-3}$	0.46	1.40	2.0–500.0

[a] Compounds: **9** – khellactone-3′,4′-di-*O*-acetyl ester, **11** – khellactone 4′-*O*-angeloyl ester (d-laserpitin), **12** – khellactone 3′-*O*-acetyl-4′-*O*-isobutyroyl ester (hyuganin D), **15** – khellactone 3′-*O*-isovaleroyl-4′-*O*-acetyl ester (dihydrosamidin), **17** – khellactone 3′-*O*-acetyl-4′-*O*-(2-methylbutyroyl) ester (hyuganin C), **27** – umbelliferone-7-*O*-(6′-apiosyl)-glucoside (6′-apiosylskimmin), **31** – khellactone-3′-*O*-glucoside (praeroside II), **36** – 5-*O*-caffeoylquinic acid.

Table S7. Intra- and inter-day precision, repeatability, stability and recovery for 8 compounds.

Compound	Precision intra-day (RSD%) *n* = 5	Precision inter-day (RSD%) *n* = 4	Repeatability (RSD%) *n* = 7	Stability (RSD%) *n* = 7	Recovery (%) *n* = 5
9	1.85	2.46	1.93	2.11	97.34
11	1.35	1.62	1.87	1.57	100.29
12	1.53	2.29	2.07	1.73	98.22
15	1.12	1.50	1.45	1.40	99.32
17	1.27	1.94	2.16	2.25	102.64
27	0.97	1.26	1.21	1.52	100.07
31	2.06	2.57	2.59	2.86	101.39
36	1.62	2.02	1.57	2.29	101.25

[a] Compounds: **9** – khellactone-3′,4′-di-*O*-acetyl ester, **11** – khellactone 4′-*O*-angeloyl ester (d-laserpitin), **12** – khellactone 3′-*O*-acetyl-4′-*O*-isobutyroyl ester (hyuganin D), **15** – khellactone 3′-*O*-isovaleroyl-4′-*O*-acetyl ester (dihydrosamidin), **17** – khellactone 3′-*O*-acetyl-4′-*O*-(2-methylbutyroyl) ester (hyuganin C), **27** – umbelliferone-7-*O*-(6′-apiosyl)-glucoside (6′-apiosylskimmin), **31** – khellactone-3′-*O*-glucoside (praeroside II), **36** – 5-*O*-caffeoylquinic acid.

Table S8. Content of 8 compounds and sum of coumarins (ΣCou) in water-methanol mixtures (WM, *v*/*v*, %) media after extraction of *Phlojodicarpus sibiricus* roots and herb, μg/mL ± S.D.

**Compound** [a]	**WM 100:0**	**WM 80:20**	**WM 60:40**	**WM 40:60**	**WM 20:80**	**WM 0:100**
			P. sibiricus roots			
9	Tr.	32.94 ± 0.65	64.56 ± 1.28	83.17 ± 1.74	95.18 ± 1.90	85.44 ± 1.69
11	Tr.	25.94 ± 0.49	83.67 ± 1.67	155.54 ± 2.94	166.22 ± 3.25	162.08 ± 3.24
12	Tr.	Tr.	Tr.	47.73 ± 0.95	52.38 ± 1.04	49.45 ± 0.97
15	30.19 ± 0.60	370.44 ± 7.41	1537.05 ± 30.74	2644.33 ± 44.95	2825.09 ± 56.50	2742.51 ± 49.35
17	Tr.	Tr.	Tr.	17.41 ± 0.31	18.75 ± 0.35	17.44 ± 0.34
27	234.22 ± 3.98	238.52 ± 4.05	241.09 ± 4.58	250.53 ± 5.01	252.64 ± 5.06	250.99 ± 5.00
31	Tr.	Tr.	Tr.	42.76 ± 0.85	46.38 ± 0.87	45.64 ± 0.88
36	Tr.	Tr.	Tr.	Tr.	Tr.	Tr.
ΣCou	264.41	667.84	1926.37	3176.33	3456.64	3353.55
			P. sibiricus herb			
9	Tr.	Tr.	Tr.	Tr.	Tr.	Tr.
11	3.91 ± 0.07	69.02 ± 1.24	256.62 ± 4.86	368.82 ± 6.25	378.19 ± 7.18	359.56 ± 6.82
12	Tr.	Tr.	Tr.	Tr.	Tr.	Tr.
15	7.22 ± 0.14	130.79 ± 2.35	346.36 ± 7.26	406.50 ± 7.71	412.92 ± 8.24	392.03 ± 7.44
17	Tr.	Tr.	Tr.	Tr.	Tr.	Tr.
27	32.29 ± 0.64	52.23 ± 0.99	72.67 ± 1.51	72.83 ± 1.57	73.10 ± 1.55	52.18 ± 1.02
31	271.00 ± 4.87	282.78 ± 5.64	288.52 ± 5.76	288.84 ± 5.48	289.14 ± 5.49	110.21 ± 2.09
36	65.74 ± 1.42	125.72 ± 2.51	154.29 ± 2.92	154.82 ± 2.29	151.95 ± 2.88	58.94 ± 1.17
ΣCou	314.42	534.82	964.17	1136.99	1153.35	913.98

[a] Compounds: **9** – khellactone-3′,4′-di-*O*-acetyl ester, **11** – khellactone 4′-*O*-angeloyl ester (d-laserpitin), **12** – khellactone 3′-*O*-acetyl-4′-*O*-isobutyroyl ester (hyuganin D), **15** – khellactone 3′-*O*-isovaleroyl-4′-*O*-acetyl ester (dihydrosamidin), **17** – khellactone 3′-*O*-acetyl-4′-*O*-(2-methylbutyroyl) ester (hyuganin C), **27** – umbelliferone-7-*O*-(6′-apiosyl)-glucoside (6′-apiosylskimmin), **31** – khellactone-3′-*O*-glucoside (praeroside II), **36** – 5-*O*-caffeoylquinic acid. Tr. – traces. Extraction conditions: 400 mg of plant material was extracted by 10 mL of water-methanol mixtures in ultrasonic bath at 50°C for 30 min and after centrifuging and filtering was analyzed in mc-HPC-UV.

Table S9. Content of 8 compounds and sum of coumarins (ΣCou) in water-methanol (20:80, *v*/*v*) media after extraction of *P. sibiricus* roots and herb by various type of extraction [a], μg/mL ± S.D.

Compound [b]	USE	MWAE	BWBE	RTE
		P. sibiricus roots		
9	95.18 ± 1.90	90.34 ± 1.80	83.16 ± 1.16	37.57 ± 0.75
11	166.22 ± 3.25	147.12 ± 2.64	125.11 ± 2.62	96.30 ± 2.01
12	52.38 ± 1.04	42.64 ± 0.85	38.63 ± 0.79	10.83 ± 0.18
15	2825.09 ± 56.50	2637.22 ± 47.46	2230.54 ± 40.14	1437.21 ± 28.74
17	18.75 ± 0.35	16.37 ± 0.27	10.84 ± 0.21	4.32 ± 0.08
27	252.64 ± 5.06	244.41 ± 3.91	240.31 ± 4.32	121.65 ± 2.55
31	46.38 ± 0.87	42.39 ± 0.85	38.10 ± 0.64	20.63 ± 0.43
36	Tr.	Tr.	Tr.	Tr.
ΣCou	3456.64	3177.85	2766.69	1728.51
		P. sibiricus herb		
9	Tr.	Tr.	Tr.	Tr.
11	378.19 ± 7.18	352.41 ± 7.04	308.22 ± 5.85	243.25 ± 4.86
12	Tr.	Tr.	Tr.	Tr.
15	412.92 ± 8.24	401.03 ± 7.21	384.15 ± 6.52	202.76 ± 3.63
17	Tr.	Tr.	Tr.	Tr.
27	73.10 ± 1.55	64.18 ± 1.28	53.62 ± 1.01	27.04 ± 0.48
31	289.14 ± 5.49	263.54 ± 4.48	208.10 ± 4.36	165.90 ± 3.30
36	151.95 ± 2.88	146.16 ± 2.92	127.22 ± 2.16	93.67 ± 1.59
ΣCou	1153.35	1081.16	954.09	638.95

[a] Extraction type: USE – ultrasound extraction (50°C), MWAE – microwave-assisted extraction (20°C), BWBE – boiled water bath extraction (95°C), RTE – room temperature extraction (20°C). [b] Compounds: **9** – khellactone-3′,4′-di-*O*-acetyl ester, **11** – khellactone 4′-*O*-angeloyl ester (d-laserpitin), **12** – khellactone 3′-*O*-acetyl-4′-*O*-isobutyroyl ester (hyuganin D), **15** – khellactone 3′-*O*-isovaleroyl-4′-*O*-acetyl ester (dihydrosamidin), **17** – khellactone 3′-*O*-acetyl-4′-*O*-(2-methylbutyroyl) ester (hyuganin C), **27** – umbelliferone-7-*O*-(6′-apiosyl)-glucoside (6′-apiosylskimmin), **31** – khellactone-3′-*O*-glucoside (praeroside II), **36** – 5-*O*-caffeoylquinic acid. Tr. – traces. Extraction conditions: 400 mg of plant material was extracted by 10 mL of water-methanol (20:80, *v*/*v*) for 30 min and after centrifuging and filtering was analyzed in mc-HPC-UV.

Table S10. Content of compounds **9**, **11**, **12**, **15**, **17**, **27**, **31**, **i**-**xi** and sum of coumarins (ΣCou) in roots of 17 *Phlojodicarpus* samples, mg/g of dry plant weight.

Compd [a]	***P. sibiricus* (S)**											
	**SY1**	**SY2**	**SY3**	**SB1**	**SB2**	**SB3**	**SB4**	**SM1**	**SM2**	**SC1**	**SC2**	**SC3**
**9**	2.33	2.52	2.07	Tr.	Tr.	Tr.	Tr.	Tr.	Tr.	Tr.	Tr.	Tr.
**11**	3.94	4.70	3.32	Tr.	Tr.	Tr.	Tr.	1.42	1.25	20.62	21.52	19.69
**12**	0.71	1.49	0.21	1.71	0.94	1.28	2.06	Tr.	Tr.	0.72	0.54	0.95
**15**	67.08	80.14	73.14	0.40	0.42	0.59	0.22	1.90	1.27	3.37	4.49	3.17
**17**	0.39	0.53	0.94	Tr.	Tr.	Tr.	Tr.	Tr.	Tr.	Tr.	Tr.	Tr.
**27**	5.82	7.47	7.04	6.25	7.16	6.33	5.11	7.63	8.11	2.78	2.04	2.11
**31**	1.55	1.39	1.30	1.59	1.63	1.50	1.11	1.53	0.97	0.41	0.35	0.30
**i**	Tr.	Tr.	Tr.	0.32	0.22	0.12	0.35	0.08	0.02	Tr.	Tr.	Tr.
**ii**	Tr.	Tr.	Tr.	4.17	4.27	5.16	2.39	3.39	4.28	0.75	1.27	1.30
**iii**	Tr.	Tr.	Tr.	4.79	5.83	7.59	2.10	2.95	3.16	0.42	0.12	0.10
**iv**	Tr.	Tr.	Tr.	1.20	0.33	0.57	0.52	0.43	0.51	Tr.	Tr.	Tr.
**v**	Tr.	Tr.	Tr.	1.56	0.37	1.27	1.09	Tr.	Tr.	Tr.	Tr.	Tr.
**vi**	Tr.	Tr.	Tr.	3.35	3.94	4.12	4.57	2.12	3.57	0.22	0.26	0.41
**vii**	Tr.	Tr.	Tr.	8.39	9.37	8.15	8.73	1.82	1.63	0.28	0.04	0.35
**viii**	Tr.	Tr.	Tr.	7.11	7.00	5.25	5.14	2.65	2.84	Tr.	Tr.	Tr.

**ix**	N.d.	N.d.	N.d.	N.d.	N.d.	N.d.	N.d.	N.d.	N.d.	N.d.	N.d.	N.d.
**x**	N.d.	N.d.	N.d.	N.d.	N.d.	N.d.	N.d.	N.d.	N.d.	N.d.	N.d.	N.d.
**xi**	N.d.	N.d.	N.d.	N.d.	N.d.	N.d.	N.d.	N.d.	N.d.	N.d.	N.d.	N.d.
ΣCou	81.82	98.24	88.02	40.84	41.48	41.93	33.39	25.92	27.61	29.57	30.63	28.38

**Compd** [a]	***P. villosus* (V)**		***P. turczaninovii* (T)**		
	**VY1**	**VB1**	**TT1**	**TT2**	**TB1**
**9**	Tr.	Tr.	0.39	0.45	0.94
**11**	7.63	8.24	0.34	0.32	0.14
**12**	2.46	2.06	N.d.	N.d.	N.d.
**15**	2.12	1.37	6.09	6.37	8.22
**17**	Tr.	Tr.	N.d.	N.d.	N.d.
**27**	17.71	21.15	3.90	4.63	3.12
**31**	1.74	1.59	1.45	0.32	1.67
**i**	Tr.	Tr.	Tr.	Tr.	Tr.
**ii**	0.62	0.34	Tr.	Tr.	Tr.
**iii**	Tr.	Tr.	1.24	0.87	1.10
**iv**	1.62	1.73	1.27	1.20	0.63
**v**	1.28	0.94	1.09	1.63	1.50
**vi**	2.62	3.15	0.53	0.28	0.11
**vii**	12.22	14.67	0.39	0.26	0.51
**viii**	2.73	3.75	Tr.	Tr.	Tr.
**ix**	N.d.	N.d.	23.90	25.63	30.47
**x**	N.d.	N.d.	24.03	21.05	32.15
**xi**	N.d.	N.d.	12.80	14.89	12.07
ΣCou	52.75	58.99	77.42	44.90	92.63

[a] Compounds: **9** – khellactone-3′,4′-di-*O*-acetyl ester, **11** – khellactone 4′-*O*-angeloyl ester (d-laserpitin), **12** – khellactone 3′-*O*-acetyl-4′-*O*-isobutyroyl ester (hyuganin D), **15** – khellactone 3′-*O*-isovaleroyl-4′-*O*-acetyl ester (dihydrosamidin), **17** – khellactone 3′-*O*-acetyl-4′-*O*-(2-methylbutyroyl) ester (hyuganin C), **27** – umbelliferone-7-*O*-(6′-apiosyl)-glucoside (6′-apiosylskimmin), **31** – khellactone-3′-*O*-glucoside (praeroside II), **36** – 5-*O*-caffeoylquinic acid. Tr. – traces. N.d. – not detected. Compounds numbered as **i**–**xi** – unidentified compounds.

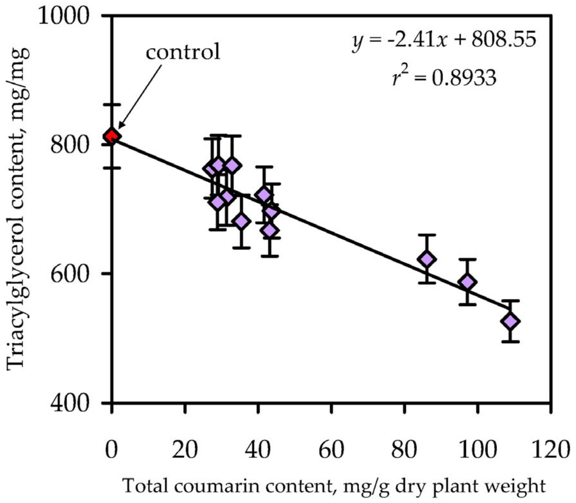

Figure S1. Correlation graph between total coumarin content (mg/g dry plant weight) in 12 samples of *Phlojodicarpus sibiricus* roots and their influence on tiacylglycerol (TG) content in 3T3-L1 adipocytes (μg/mg protein). The following data in Table used to build the graph.

Table for Figure S1. Total coumarins content (TCC) in *P. sibiricus* roots and influence of *Phlojodicarpus* extracts on tiacylglycerol (TG) content in 3T3-L1 adipocytes [1].

Sample no	TCC, mg/g [a,2]	TG, μg/mg protein [b,3]
SY1 (50 μg/mL)	86.14 ± 1.72	622.7 ± 24.9 *
SY2 (50 μg/mL)	108.94 ± 1.96	526.4 ± 20.5 *
SY3 (50 μg/mL)	97.20 ± 1.74	587.3 ± 19.9 *
SB1 (50 μg/mL)	41.63 ± 0.71	722.1 ± 28.8 *
SB2 (50 μg/mL)	43.15 ± 0.69	667.2 ± 22.6 *
SB3 (50 μg/mL)	43.76 ± 0.92	697.4 ± 23.6 *
SB4 (50 μg/mL)	35.37 ± 0.64	681.4 ± 29.2 *
SM1 (50 μg/mL)	27.54 ± 0.55	763.1 ± 31.2
SM2 (50 μg/mL)	29.18 ± 0.59	768.2 ± 30.7
SC1 (50 μg/mL)	31.40 ± 0.56	718.4 ± 25.8 *
SC2 (50 μg/mL)	32.89 ± 0.51	767.6 ± 29.9
SC3 (50 μg/mL)	28.96 ± 0.43	711.0 ± 22.7 *
Control	-	812.8 ± 25.1
5CQA (10 μg/mL)	-	286.2 ± 11.45 *

[1] Averages ± standard deviation were obtained from three ([a]) or four ([b]) different experiments. [2] Dry extract weight. [3] Values with asterisk (*) indicate statistically significant differences with the control groups at $p < 0.05$ by one-way ANOVA.

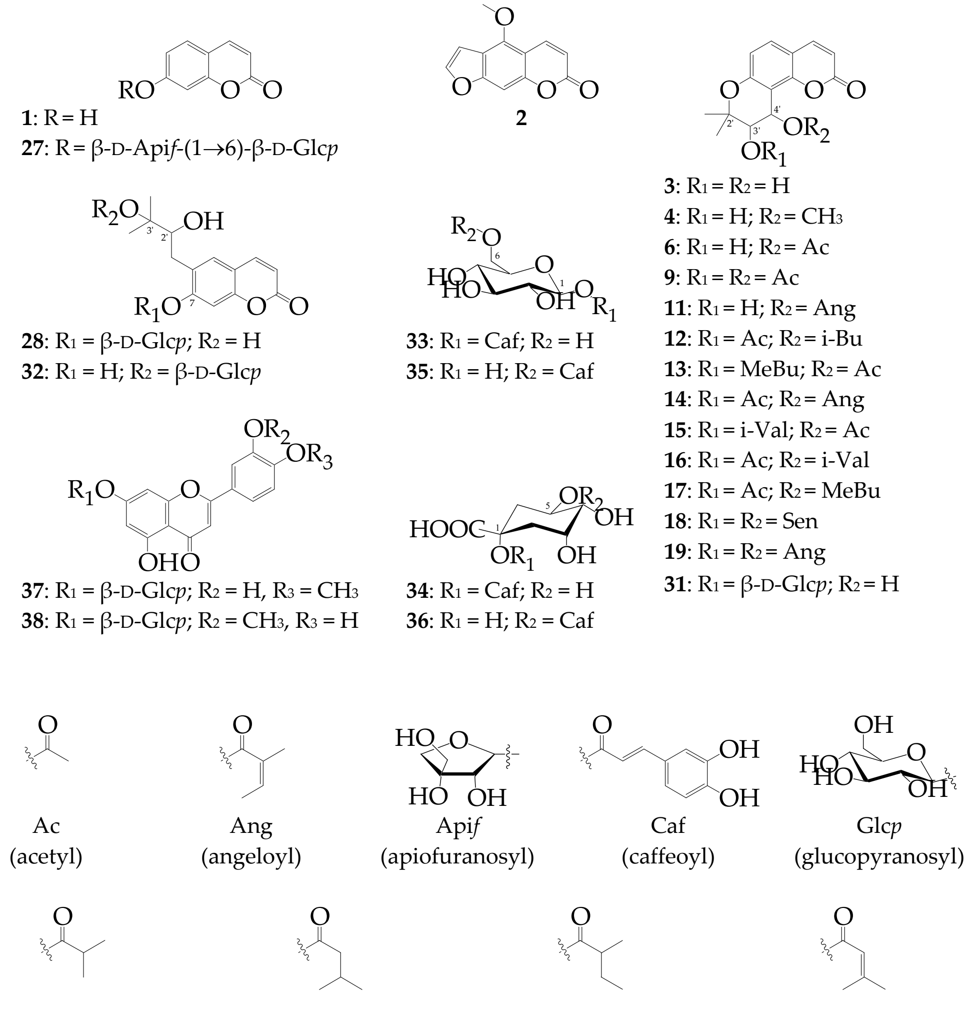

i-Bu	i-Val	MeBu	Sen
(isobutyroyl)	(isovaleroyl)	(2-methylbutyroyl)	(senecioyl)

Figure S2. Structures of reference standards used in present work. Abbreviations structures showed below.

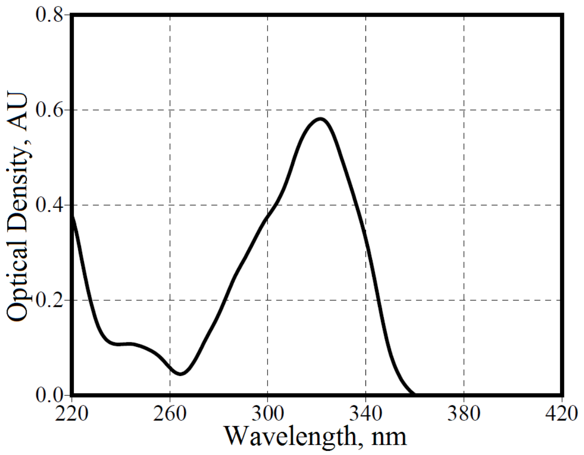

Type A (dihydrosamidin)

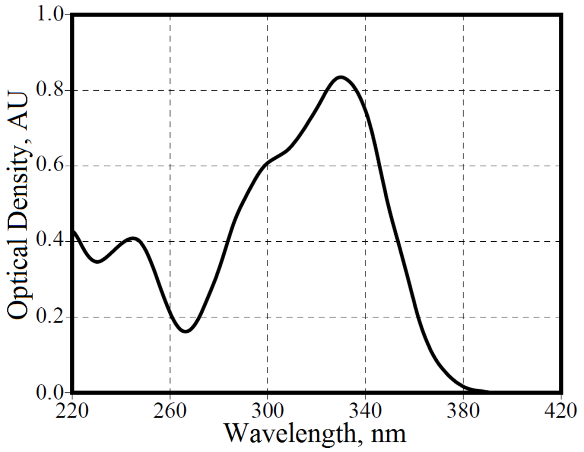

Type B (5-*O*-caffeoylquinic acid)

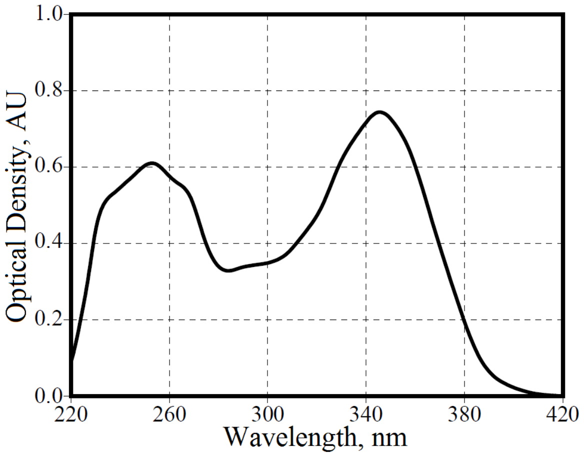

Type C (diosmetin-7-*O*-glucoside)

Figure S3. Types of UV spectral patterns.

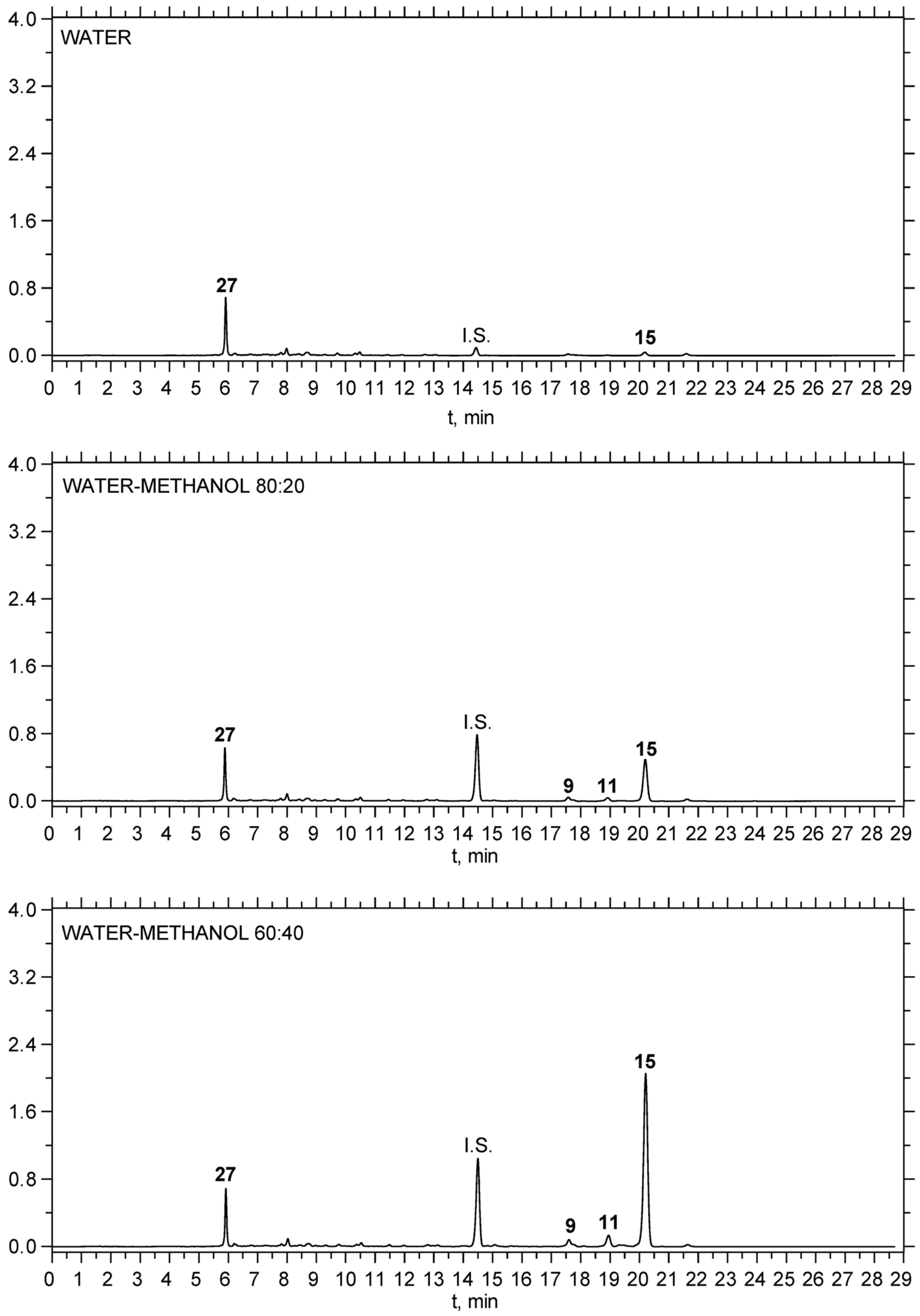
WATER
WATER-METHANOL 80:20
WATER-METHANOL 60:40
27
I.S.
9
11
15
t, min
4.0
3.2
2.4
1.6
0.8
0.0
0 1 2 3 4 5 6 7 8 9 10 11 12 13 14 15 16 17 18 19 20 21 22 23 24 25 26 27 28 29

Figure S4. *Cont*.

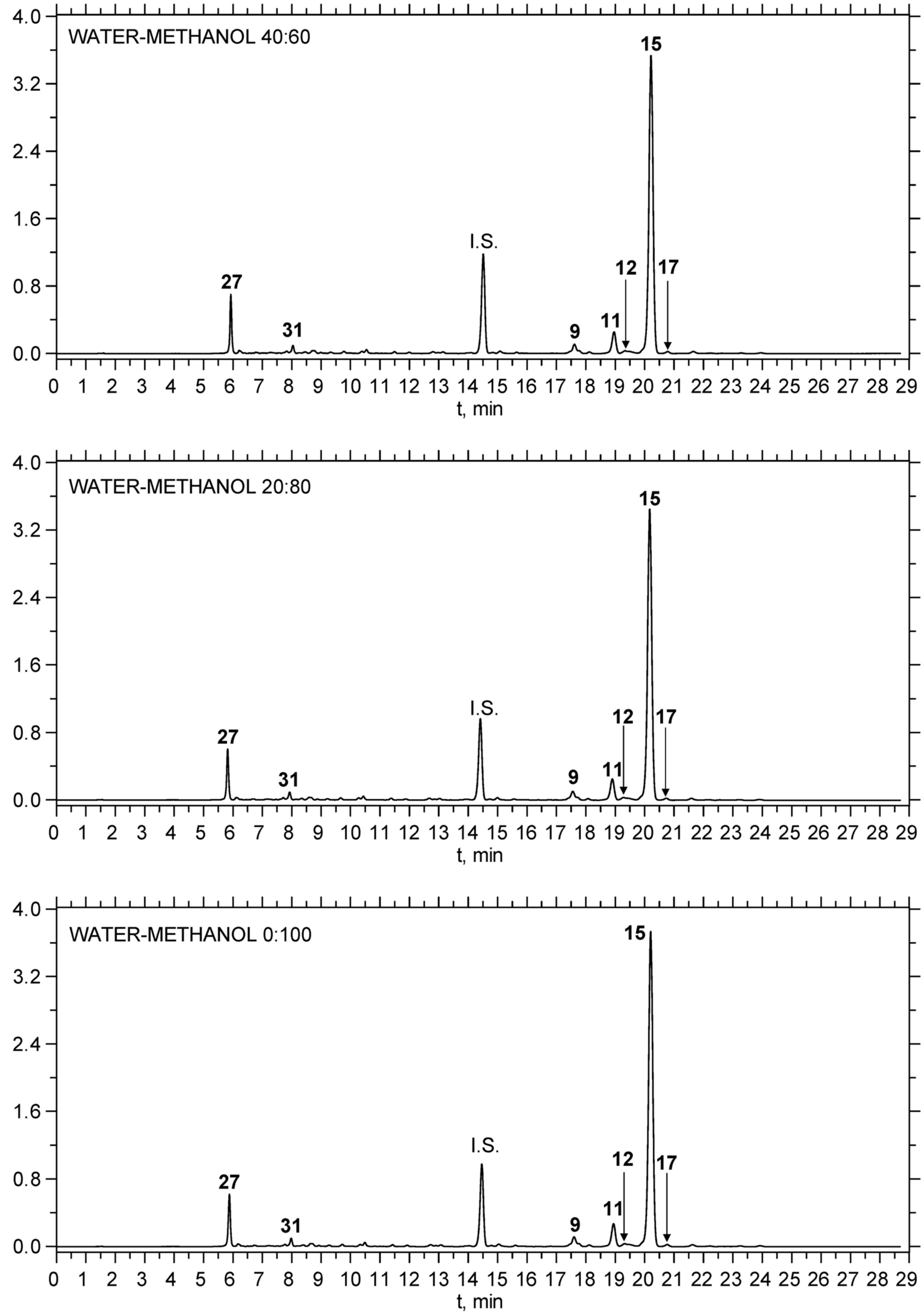

Figure S4. mcHPLC-UV chromatograms of *P. sibiricus* roots extracts prepared with various solvents. Compounds numbered as **9** – khellactone-3′,4′-di-*O*-acetyl ester, **11** – khellactone 4′-*O*-angeloyl ester (d-laserpitin), **12** – khellactone 3′-*O*-acetyl-4′-*O*-isobutyroyl ester (hyuganin D), **15** – khellactone 3′-*O*-isovaleroyl-4′-*O*-acetyl ester (dihydrosamidin), **17** – khellactone 3′-*O*-acetyl-4′-*O*-(2-methylbutyroyl) ester (hyuganin C), **27** – umbelliferone-7-*O*-(6′-apiosyl)-glucoside (6′-apiosylskimmin), **31** – khellactone-3′-*O*-glucoside (praeroside II). I.S. – internal standard (pimpinellin).

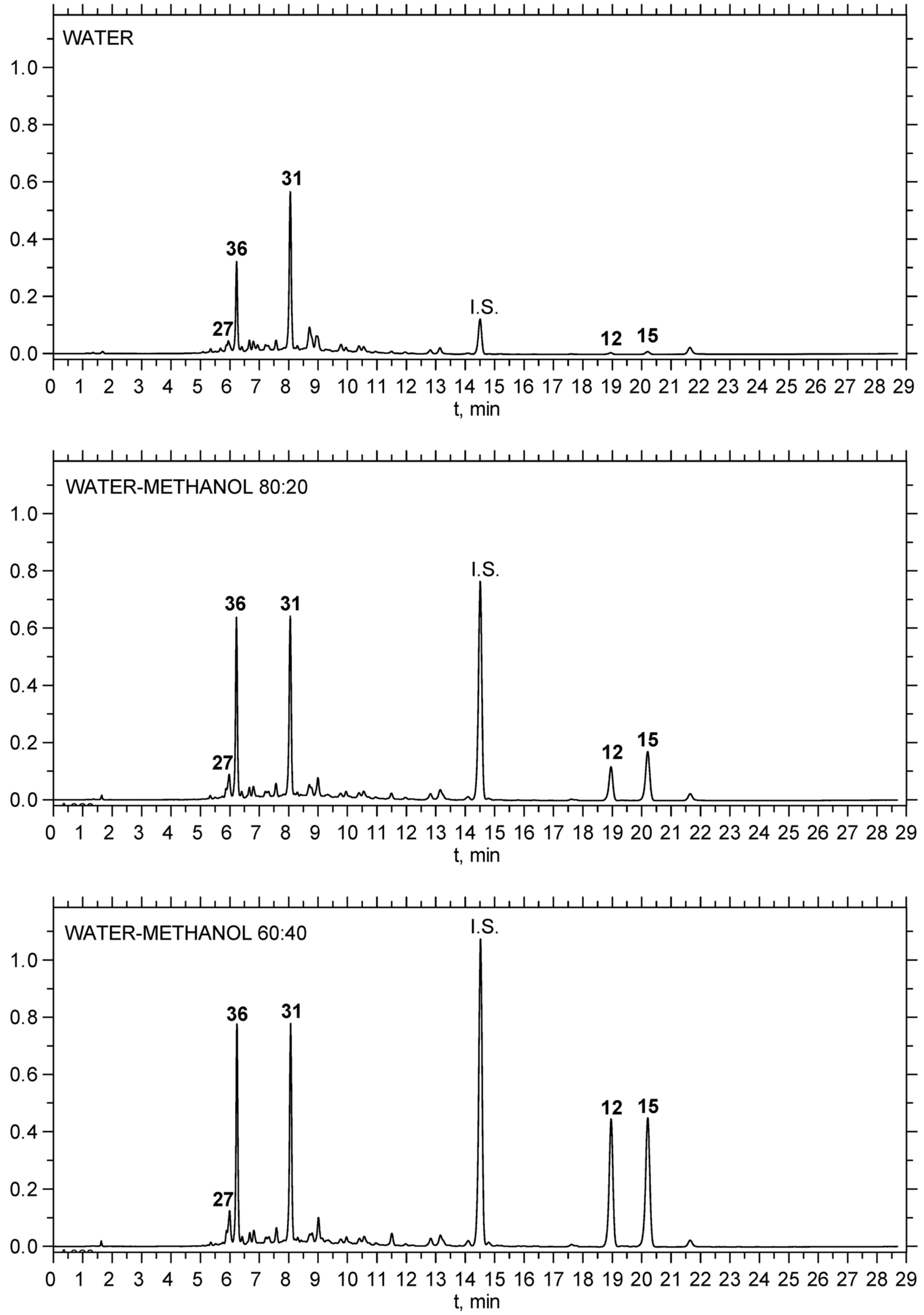
WATER
36
31
27
I.S.
12
15
t, min
WATER-METHANOL 80:20
36
31
27
I.S.
12
15
t, min
WATER-METHANOL 60:40
36
31
27
I.S.
12
15
t, min

Figure S5. *Cont.*

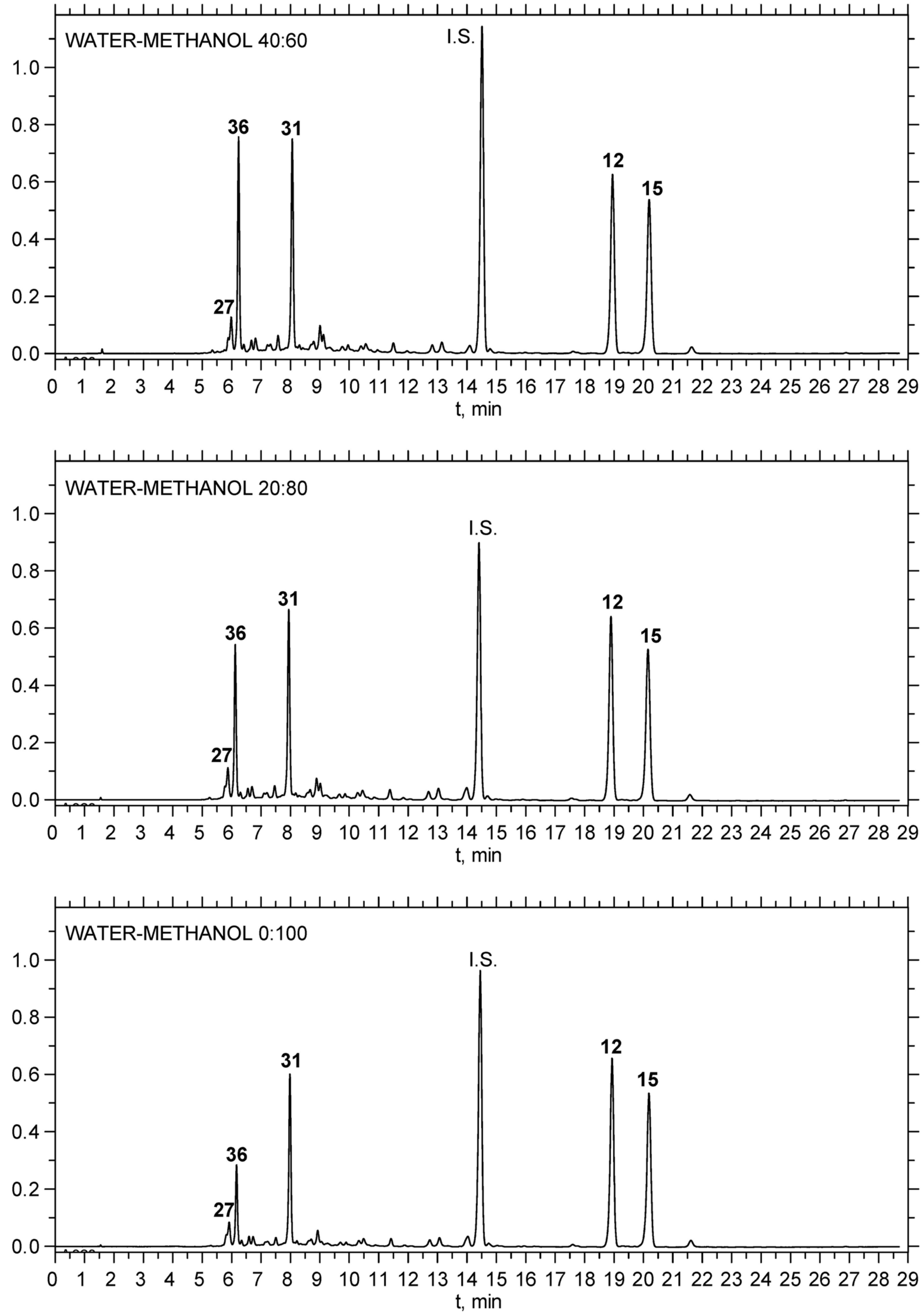

Figure S5. mcHPLC-UV chromatograms of *P. sibiricus* herb extracts prepared with various solvents. Compounds numbered as **11** – khellactone 4′-*O*-angeloyl ester (d-laserpitin), **15** – khellactone 3′-*O*-isovaleroyl-4′-*O*-acetyl ester (dihydrosamidin), **27** – umbelliferone-7-*O*-(6′-apiosyl)-glucoside (6′-apiosylskimmin), **31** – khellactone-3′-*O*-glucoside (praeroside II), **36** – 5-*O*-caffeoylquinic acid. I.S. – internal standard (pimpinellin).

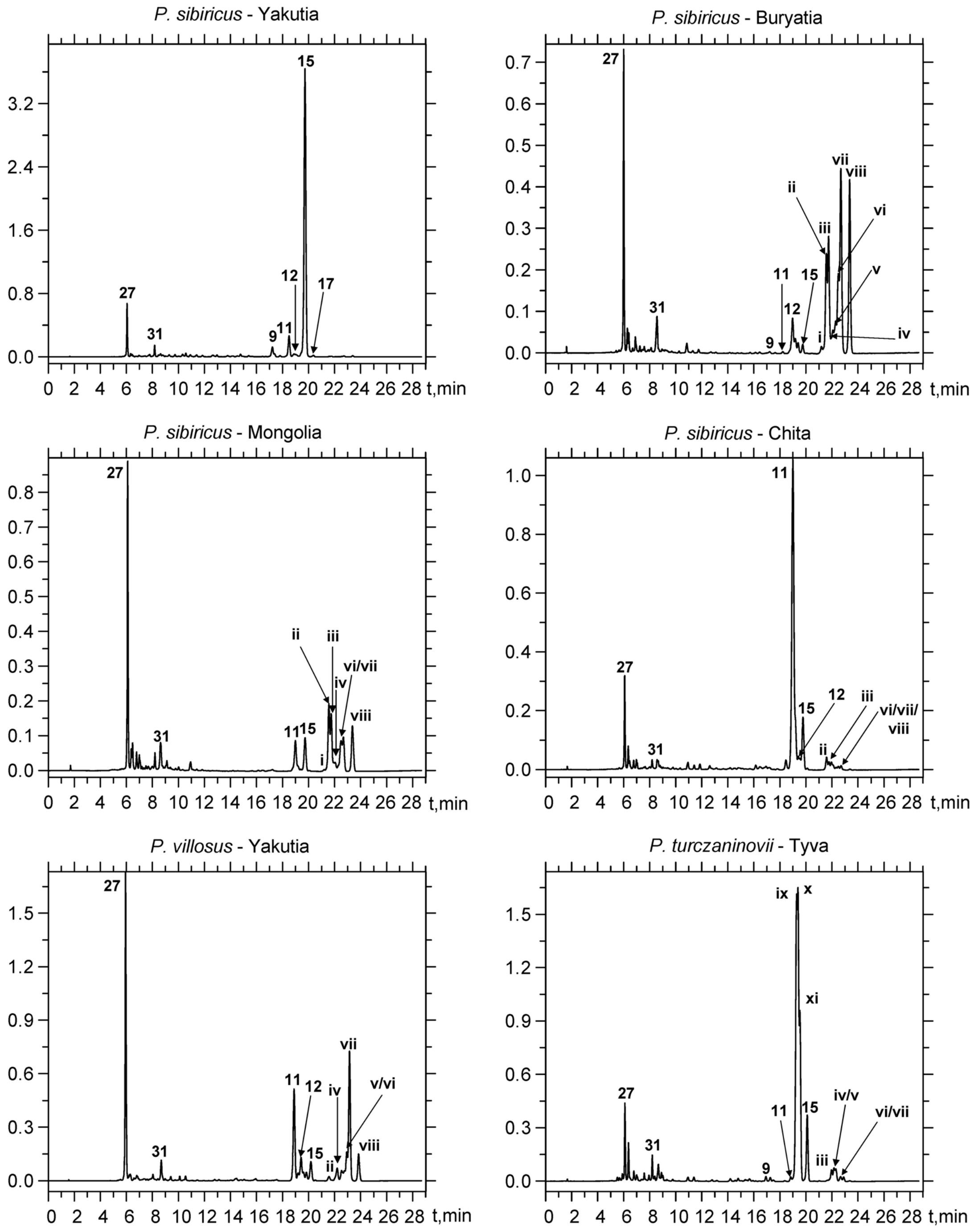

Figure S6. mc-HPLC-UV chromatograms of *Phlojodicarpus* root extracts from various regions. Compounds numbered as **9** – khellactone-3′,4′-di-*O*-acetyl ester, **11** – khellactone 4′-*O*-angeloyl ester (d-laserpitin), **12** – khellactone 3′-*O*-acetyl-4′-*O*-isobutyroyl ester (hyuganin D), **15** – khellactone 3′-*O*-isovaleroyl-4′-*O*-acetyl ester (dihydrosamidin), **17** – khellactone 3′-*O*-acetyl-4′-*O*-(2-methylbutyroyl) ester (hyuganin C), **27** – umbelliferone-7-*O*-(6′-apiosyl)-glucoside (6′-apiosylskimmin), **31** – khellactone-3′-*O*-glucoside (praeroside II). Compounds numbered as **i–xi** – unidentified compounds.
